# Genetic determinants of IgG antibody response to COVID-19 vaccination

**DOI:** 10.1016/j.ajhg.2023.12.005

**Published:** 2024-01-04

**Authors:** Shengzhe Bian, Xinxin Guo, Xilai Yang, Yuandan Wei, Zijing Yang, Shiyao Cheng, Jiaqi Yan, Yongkun Chen, Guo-Bo Chen, Xiangjun Du, Stephen S. Francis, Yuelong Shu, Siyang Liu

**Affiliations:** 1 School of Public Health (Shenzhen), Shenzhen Campus of Sun Yat-sen University, Shenzhen 518107, P.R. China; 2 School of Public Health (Shenzhen), Sun Yat-sen University, Guangzhou 510006, P.R. China; 3 Center for General Practice Medicine, Department of General Practice Medicine, Clinical Research Institute, Zhejiang Provincial People’s Hospital, People’s Hospital of Hangzhou Medical College, Hangzhou 310059, Zhejiang, P.R. China; 4 Key Laboratory of Endocrine Gland Diseases of Zhejiang Province, Hangzhou 310063, Zhejiang, P.R. China; 5 Key Laboratory of Tropical Disease Control, Ministry of Education, Sun Yat-sen University, Guangzhou 510006, P.R. China; 6 Department of Epidemiology and Biostatistics, University of California, San Francisco, San Francisco, CA 94143, USA; 7 Department of Neurological Surgery, University of California, San Francisco, San Francisco, CA 94143, USA; 8 Helen Diller Family Comprehensive Cancer Center, University of California, San Francisco, San Francisco, CA 94143, USA; 9 Weill Institute for Neurosciences, University of California, San Francisco, San Francisco, CA 94158, USA; 10 Institute of Pathogen Biology, Chinese Academy of Medical Sciences & Peking Union Medical College, Beijing 102629, P.R. China

**Keywords:** IgG response to vaccines, COVID-19, human leukocyte antigen, genome-wide association study, transcriptome-wide association study, fine-mapping, cross-ancestry, electrostatic potential energy

## Abstract

Human humoral immune responses to SARS-CoV-2 vaccines exhibit substantial inter-individual variability and have been linked to vaccine efficacy. To elucidate the underlying mechanism behind this variability, we conducted a genome-wide association study (GWAS) on the anti-spike IgG serostatus of UK Biobank participants who were previously uninfected by SARS-CoV-2 and had received either the first dose (n = 54,066) or the second dose (n = 46,232) of COVID-19 vaccines. Our analysis revealed significant genome-wide associations between the IgG antibody serostatus following the initial vaccine and human leukocyte antigen (HLA) class II alleles. Specifically, the *HLA-DRB1^∗^13:02* allele (MAF = 4.0%, OR = 0.75, p = 2.34e−16) demonstrated the most statistically significant protective effect against IgG seronegativity. This protective effect was driven by an alteration from arginine (Arg) to glutamic acid (Glu) at position 71 on HLA-DRβ1 (p = 1.88e−25), leading to a change in the electrostatic potential of pocket 4 of the peptide binding groove. Notably, the impact of HLA alleles on IgG responses was cell type specific, and we observed a shared genetic predisposition between IgG status and susceptibility/severity of COVID-19. These results were replicated within independent cohorts where IgG serostatus was assayed by two different antibody serology tests. Our findings provide insights into the biological mechanism underlying individual variation in responses to COVID-19 vaccines and highlight the need to consider the influence of constitutive genetics when designing vaccination strategies for optimizing protection and control of infectious disease across diverse populations.

## Graphical abstract

**Figure:**
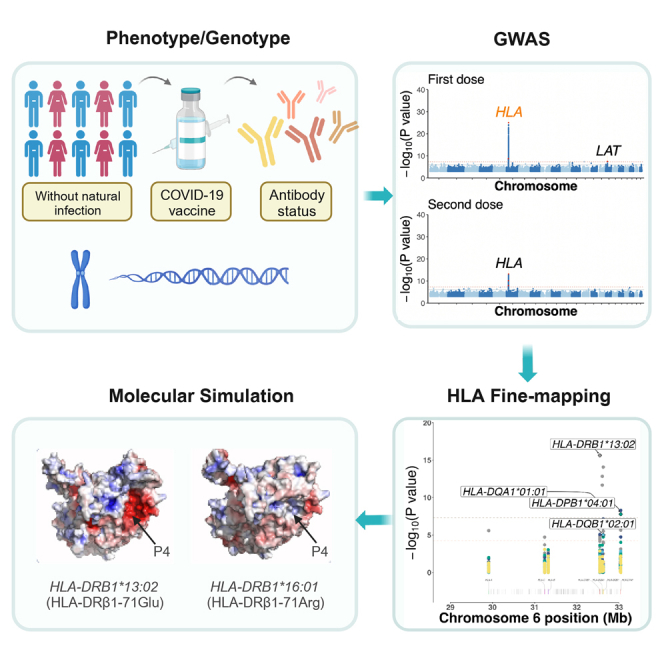

## Teaser

Antibody production after COVID-19 vaccination is closely related to vaccine efficacy. Our findings about its host genetic determinants provide insights into the biological mechanism of host responses to COVID-19 vaccines and have guiding significance for vaccine design and vaccination.

## Introduction

Coronavirus disease 2019 (COVID-19), caused by the severe acute respiratory syndrome coronavirus 2 (SARS-CoV-2), has emerged as a global pandemic, posing a significant and enduring threat to public health.^1^^,^^2^ As of November 2023, there have been more than 772 million confirmed cases worldwide, with the death toll surpassing 6 million (https://covid19.who.int/). Vaccination has emerged as an effective strategy for protecting high-risk populations. The primary approach of COVID-19 vaccines involves utilizing the spike protein of the virus as an antigen to stimulate the human immune response.^3^ By May 2022, 57 countries had successfully vaccinated up to 70% of their population (https://www.who.int/director-general/speeches/detail/who-director-general-s-opening-address-at-the-75th-world-health-assembly---22-may-2022). However, the effectiveness of the vaccine has shown variation, leaving room for improvement. For instance, the ChAdOx1 vaccine, widely administered in the United Kingdom (UK), exhibits a vaccine efficacy of 62.1% (95% CI 41.0–75.7) after two standard doses (vaccine efficacy was calculated as 1 − relative risk derived from a robust Poisson regression model adjusted for age).^4^ One crucial method to enhance vaccine efficacy is to understand the biological mechanisms underlying the host immune responses, specifically the host immunogenicity.^5^ Host immunogenicity can differ among individuals, even for the same vaccine, in terms of production, potency, and duration of antibodies.^6^ Notably, host genetic factors, along with variables such as sex, age, and ethnicity, have been identified as significant contributors to personal immunogenicity through twin experiments.^7^ Therefore, comprehending the host genetics perspective and its influence on the antibody response to COVID-19 vaccines is imperative and holds promise for meaningful insights into the immuno-pathogenesis of COVID-19, vaccine development, and potential immunotherapies.

Host immune response to certain vaccines constitutes a multifaceted process involving humoral and/or cell-mediated immune reactions. Previous research has identified genetic factors implicated in pathways such as antigen processing and presentation,^8^^,^^9^^,^^10^ innate recognition receptors, and cellular signaling^11^^,^^12^^,^^13^ that influence immune responses to vaccines such as smallpox, rubella, measles, hepatitis, and influenza. However, due to financial and logistical challenges, many of these studies have difficulty distinguishing whether antibodies originate from natural infection or vaccines. In addition, these studies were often constrained by limited sample sizes and a focus on specific ethnic or population groups which potentially introduced bias in estimating genetic effect, thus limiting the generalizability of the findings to broader populations.

Similar limitations are observed in studies examining the host response to COVID-19 vaccination. A genome-wide association study (GWAS) of antibody levels in 168 recipients of inactivated SARS-CoV-2 vaccine identified a total of 177 significant SNPs corresponding to 41 independent loci.^14^ In targeted studies of the human leukocyte antigen (HLA), no association was found with anti-spike IgG levels (n = 78 for first-dose vaccination).^15^ A recent GWAS of 1,076 participants in the UK identified two genetic associations, specifically *HLA-DQB1^∗^06* and HLA-DRβ1-71Glu/Arg, with antibody levels after a single dose of vaccination.^16^ However, these studies may be susceptible to potential confounding by prior natural infection. The limited sample size or lack of replication in these studies restrict the generalizability of these results to the general population. Moreover, given the high mutation rate and complex linkage disequilibrium patterns of genes involved in immune responses, such as killer cell immunoglobulin-like receptors (KIR) and human leukocyte antigen (HLA) genes, clarifying the relationship between these genes and immune responses necessitates sophisticated fine-mapping and functional genomic analytical strategies. Furthermore, whether the genetic basis for host immunogenicity to specific vaccines may overlap with the risk of infection or adverse outcomes remains an unknown question that requires in-depth investigation among large-scale cohorts with comprehensive phenotypes.

The UK Biobank (UKBB) has taken swift strides to help tackle the global pandemic by undertaking five major initiative studies. In this study, we designed and leveraged serum antibody status data of spike (S) and nucleocapsid (N) proteins from two studies: the Coronavirus self-test antibody study, which involved 200K participants, and the Coronavirus infection study, which involved 60K participants, along with the genetic and health linkage data from UKBB. The availability of both S and N protein serum antibody status for the participants allows us to exclude previously SARS-CoV-2-infected individuals and dissect the genuine genetic effects associated with vaccine response while mitigating the confounding effects of prior SARS-CoV-2 infection. The availability of two different types of antibody tests for anti-spike IgG serum status allows us to classify participants into three study cohorts (discovery, replication, and combined cohort) for conducting the GWAS and replication. The comprehensive health linkage data from UKBB further enables the investigation of the genetic correlation between IgG response and health outcomes. We focus on presenting consistent results from the two independent cohorts and biological insights into the host genetic factors influencing the host immune response to COVID-19 vaccination.

## Subjects and methods

### Phenotype definitions

In February 2022, the UK Biobank (UKBB) released two study datasets: the 200K self-test antibody study (UKBB show case Category 998) and the 60K Coronavirus infection study (UKBB show case Category 997). The first study used antibody lateral flows tests (LFTs) for at-home testing spike protein immunoglobulin G (IgG) antibodies. Phase 1 of the study recruited about 50,000 participants who had previously attended a baseline UK Biobank imaging assessment using Fortress Fast test kit. In Phase 2, an additional 150,000 participants were recruited using the AbC-19 Rapid Test. The second study served as a follow-on study. Participants who tested seropositive and had been vaccinated in the first study (the 200K self-test antibody study) were invited to submit a post-capillary blood sample to the Thriva laboratory to detect nucleocapsid antibodies using the Elecsys Anti-SARS-CoV-2 immunoassay. This assay distinguishes between antibodies produced as a result of infection and those produced following vaccination, utilizing a recombinant protein representing the nucleocapsid (N) antigen in a double-antigen sandwich assay format, favoring the detection of high-affinity antibodies against SARS-CoV-2. The clinical sensitivity of this test method in identifying new coronavirus infection is 99.5% (95% CI: 97.0–100) more than 14 days after PCR confirmation, with a clinical specificity of 99.8% (95% CI: 99.69–99.88) (https://diagnostics.roche.com/gb/en/products/params/elecsys-anti-sars-cov-2.html).

**Figure 1:**
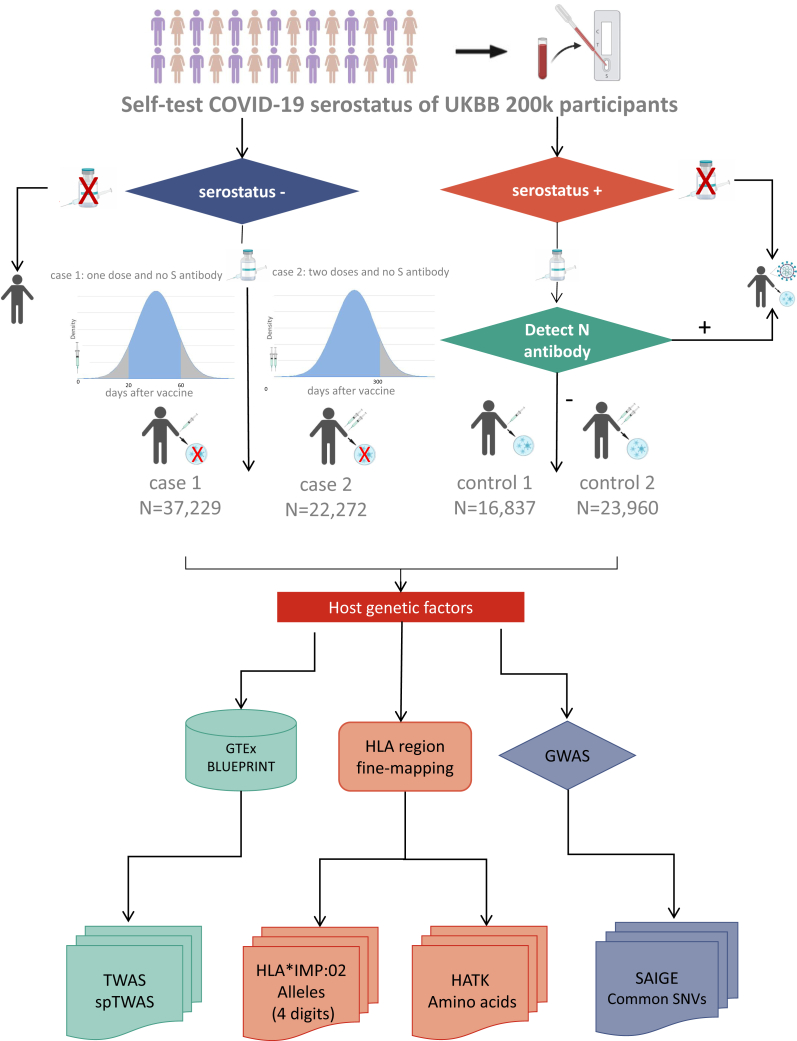
Flow chart describing phenotype definition and primary analyses The study involved 200K participants who participated in the self-test study for SARS-CoV-2 spike protein antibody (S antibody). Phenotype categorization was based on S antibody negativity and the administration of the first or second doses of vaccination, with the interval between vaccination and antibody detection falling within 20–60 days (0–300 days for the second-dose vaccination). Case 1 refers to individuals who tested seronegative for S-antibodies after receiving one dose of the vaccine, while case 2 pertains to those who tested seronegative after two doses of the vaccine. The sample size was determined from the combined cohort. Notably, individuals testing S antibody negative without vaccination were classified as not having undergone the immunization process. Regarding the control group, individuals with a positive S antibody test result and who received one or two doses of the vaccine were included. However, further testing for nucleocapsid antibodies (N antibodies) was performed, and those with a negative N antibody result were defined as control 1 (seropositive after one dose of vaccine) and control 2 (seropositive after two doses of vaccine). These individuals were deemed to have produced antibodies as a result of vaccination. Notably, individuals who tested positive for S-antibodies without vaccination or tested positive for both S and N antibodies were excluded from the phenotype definition, as they were considered to have been infected with COVID-19. The sample size is calculated from the combined cohort. After the phenotype definition, subsequent analyses focused on identifying host genetic factors. These analyses included genome-wide association studies (GWASs) involving common single variants and rare variants, association analysis of alleles and amino acids following HLA region imputation, as well as transcriptome-wide association studies (TWASs) and single-cell splicing transcriptome-wide association studies (spTWASs) utilizing data from the GTEx and BLUEPRINT projects.

To examine the host response to COVID-19 vaccines, we defined two phenotypes: host serostatus after the first and second vaccine doses. To account for variations in the timing of antibody production following vaccination, we based our approach on documented IgG production time frame after the first and second vaccine doses as described in previous literature.^17^ We limited the cases to individuals with a negative IgG serostatus between 20 and 60 days after their first vaccine dose and a negative IgG serostatus within 0–300 days after their second vaccine dose, as determined by LFTs. Notably, LFTs detect only spike antibodies (which result from both infection and vaccination), while nucleocapsid antibodies are exclusively produced in response to infection. Therefore, we defined true vaccine-induced seropositivity after vaccination as positive LFT result with a negative nucleocapsid antibody test. The definition of the affected individuals and control subjects for the two phenotypes follows the criteria presented in Figure 1, with detailed phenotype definitions provided in Table S1.

(1) After the first dose of the vaccine, affected individuals (seronegative for the first dose vaccination, n = 45,400) were defined as those who had received only the first dose of the vaccine (UKB field ID 27983 and 27985), whose LFTs results were negative (UKB field ID 27981), and whose time between conducting LFTs and receiving the first-dose vaccination was within 20–60 days (UKB field ID 27982 and 27984). Control subjects (seropositive for the first dose vaccination, n = 20,176) were defined as those who had received only the first dose of the vaccine, whose LFTs results were positive, and whose nucleocapsid antibodies test results were negative (UKB field ID 27990).
(2) After the second dose of the vaccine, affected individuals (seronegative for the second dose vaccination, n = 27,281) were defined as those who had received the first dose vaccine (UKB field ID 27985), whose LFTs results were negative, and whose time between conducting LFTs and receiving the second dose vaccination was within 0–300 days (UKB field ID 27982 and 27986). Control subjects (seropostive for the second dose vaccination, n = 28,976) were defined as those who had received the second dose of the vaccine, whose LFTs results were positive, and whose nucleocapsid antibodies test results were negative (UKB field ID 27990).

All study participants provided informed consent. For this study, data from the UKB resource were accessed under the application number 41376. The protocols governing the use of UKB data are overseen by The UK Biobank Ethics Advisory Committee (EAC). For more information, see https://www.ukbiobank.ac.uk/ethics/.

### UKBB genetic data

The UK Biobank (UKB) has genotyped for nearly 500,000 individuals. Participants were genotyped on the Affymetrix Axiom UK Biobank array (89%) or the UK BiLEVE array (11%) with genome-wide imputation performed using the Haplotype Reference Consortium release 1 data^18^ (https://imputationserver.sph.umich.edu/index.html#!) and the merged 1000 Genomes phase 3 (23 November 2010 data freezes) and UK10K reference panels,^19^^,^^20^ which are available from the European Genome-phenome archive under the accession codes EGAS00001000713 (EGA study) and EGAD00001000776 (EGA dataset) under managed access conditions (http://www.uk10k.org/data_access.html). The SNP database (dbSNP) reference SNP (rs) IDs were assigned to as many markers as possible using reference SNP ID lists available from the UCSC genome annotation database for the GRCh37 assembly of the human genome (http://hgdownload.cse.ucsc.edu/goldenpath/hg19/database/). We performed standard quality control on genetic data. For SNPs, using PLINK 2.0^21^ command --geno 0.02 --rm-dup ’exclude-all’ excluded SNPs with low genotype call rate and duplicated marker name. What’s more, we excluded SNPs with imputation quality INFO < 0.30 according to “ukb_imp_mfi” provided by UKB. Finally, imputation data retain 78,919,673 SNPs, and array data retain 695,934 SNPs. For sample quality control, we obey the following criteria: (1) use command “plink2 --mind 0.02” to filter low genotype call rate samples, (2) keep samples that are not marked as outliers for heterozygosity and missing rates (UK Biobank field ID 22027), (3) remove samples wherein the self-reported and genotype-determined sex are in discordance (UKB Field ID 31 and 22001), and (4) keep samples that have at most ten putative third-degree relatives (UKB Field ID 22021).

To minimize the variability due to population structure in our dataset, we restricted our primary analysis to white British ancestry according to UKB field ID 22006 (n = 409,616), while non-European populations (including SAS, EAS, and AFR) were a secondary analysis. In addition, we combined the available genetic data with the phenotype data and divided the two phenotypes into three study cohorts (Table 1) namely discovery, replication, and combined, according to the phase and LFTs Kits of the UKBB 200K self-test antibody study for subsequent association analysis. In order to remove related individuals in each phenotype cohort, within for each set of three-degree relatives, we keep one in every study cohort using command “plink2 --king-cutoff 0.0442.”

**Table 1:** The comparison of subject characteristics between discovery and replication cohorts for first-dose and second-dose vaccination

**Demographic characteristics**	**Combined cohort**	**Discovery cohort**	**Replication cohort**
**Serostatus after the first-dose vaccination**			

Total	54,066^a^	34,632	20,078
Age		p < 2.20e−6^c^	
Median age (IQR)^b^	54 (12)	50 (9)	62 (10)
Townsend deprivation index		p < 2.20e−16	
Median Townsend deprivation index (IQR)	−2.7 (3.19)	−2.58 (3.39)	−2.86 (2.86)
Sex		p < 2.2e−16	
Male	30,141 (55.7%)^d^	20,182 (58.3%)	10,336 (51.5%)
Female	23,925 (44.3%)	14,450 (41.7%)	9,742 (48.5%)
Country of birth		p < 2.2e−16	
England	45,647 (84.4%)	29,011 (83.8%)	17,185 (85.6%)
Scotland	4,725 (8.7%)	2,991 (8.6%)	1,803 (9.0%)
Wales	2,222 (4.1%)	1,627 (4.7%)	611 (3.0%)
Others	1,472 (2.7%)	1,003 (2.9%)	479 (2.4%)
Vaccine type		p < 2.2e−16	
ChAdOx1	19,948 (36.9%)	14,471 (41.8%)	5,682 (28.3%)
BNT1162b2	7,124 (13.2%)	2,964 (8.6%)	4,245 (21.1%)
Unknown	26,994 (49.9%)	17,197 (49.7%)	10,151 (50.6%)

**Serostatus after the second-dose vaccination**			

Total	46,232	42,011	4,414
Age		p < 2.2e−16	
Median age (IQR)	60 (8)	59 (8)	66 (3)
Townsend deprivation index		p < 0.0001468	
Median Townsend deprivation index (IQR)	−2.61 (3.29)	−2.59 (3.31)	−2.75 (3.105)
Sex		p < 2.20e−16	
Male	26,584 (57.5%)	24,452 (58.2%)	2,242 (50.8%)
Female	19,648 (42.5%)	17,559 (41.8%)	2,172 (49.2%)
Country of birth		p < 0.33	
England	39,210 (84.8%)	35,644 (84.8%)	3,732 (84.5%)
Scotland	3,291 (7.1%)	2,972 (7.1%)	330 (7.5%)
Walse	2,645 (5.7%)	2,421 (5.8%)	237 (5.4%)
Others	1,086 (2.3%)	974 (2.3%)	115 (2.6%)
Vaccine type		p < 2.20e−16	
ChAdOx1	13,233 (28.6%)	12,588 (30.0%)	683 (15.5%)
BNT1162b2	11,039 (23.9%)	9,426 (22.4%)	1,671 (37.9%)
Unknown	21,960 (47.5%)	19,997 (47.6%)	2,060 (46.7%)

a The “combined” cohort refers to the aggregation of the “discovery” and “replication” cohorts, followed by the exclusion of related individuals.

b Interquartile range.

c The p value of the t test or chi-squared test comparing the characteristics of discovery and replication cohorts.

d The number in parentheses is the proportion of that category to the total number of people in that cohort.

### Variant annotation

Variant annotation was implemented in Ensembl Variant Effect Predictor (VEP)^22^ v.106 to provide the consequences and effects of variants on transcripts, using indexed GRCh37 v.106 cache files for *Homo sapiens*. We used --pick options to pick one block of consequence for each variant according to a set of VEP default criteria. --nearest symbol option was used to identify the nearest gene symbol with a protein-coding transcription start site (TSS) of variants.

### Genome-wide association analysis

To replicate the significant SNPs of the association analysis, we divided the two phenotypes into three cohorts (Table 1)—discovery, replication and combined—and these cohorts were used separately to conduct genome-wise association analysis. The principal components of each cohort were calculated by PLINK 2.0^21^ using the array data. We selected approximately 240,000 SNPs for analysis that passed quality control with PLINK 2.0 --indep-pairwise 500 50 0.2 and --maf 0.01. We used PLINK 2.0 --pca biallelic-var-wts approx vzs for cohorts of more than 5,000 samples and PLINK 2.0 --pca biallelic-var-wts vzs for those fewer than 5,000.

**Figure 2:**
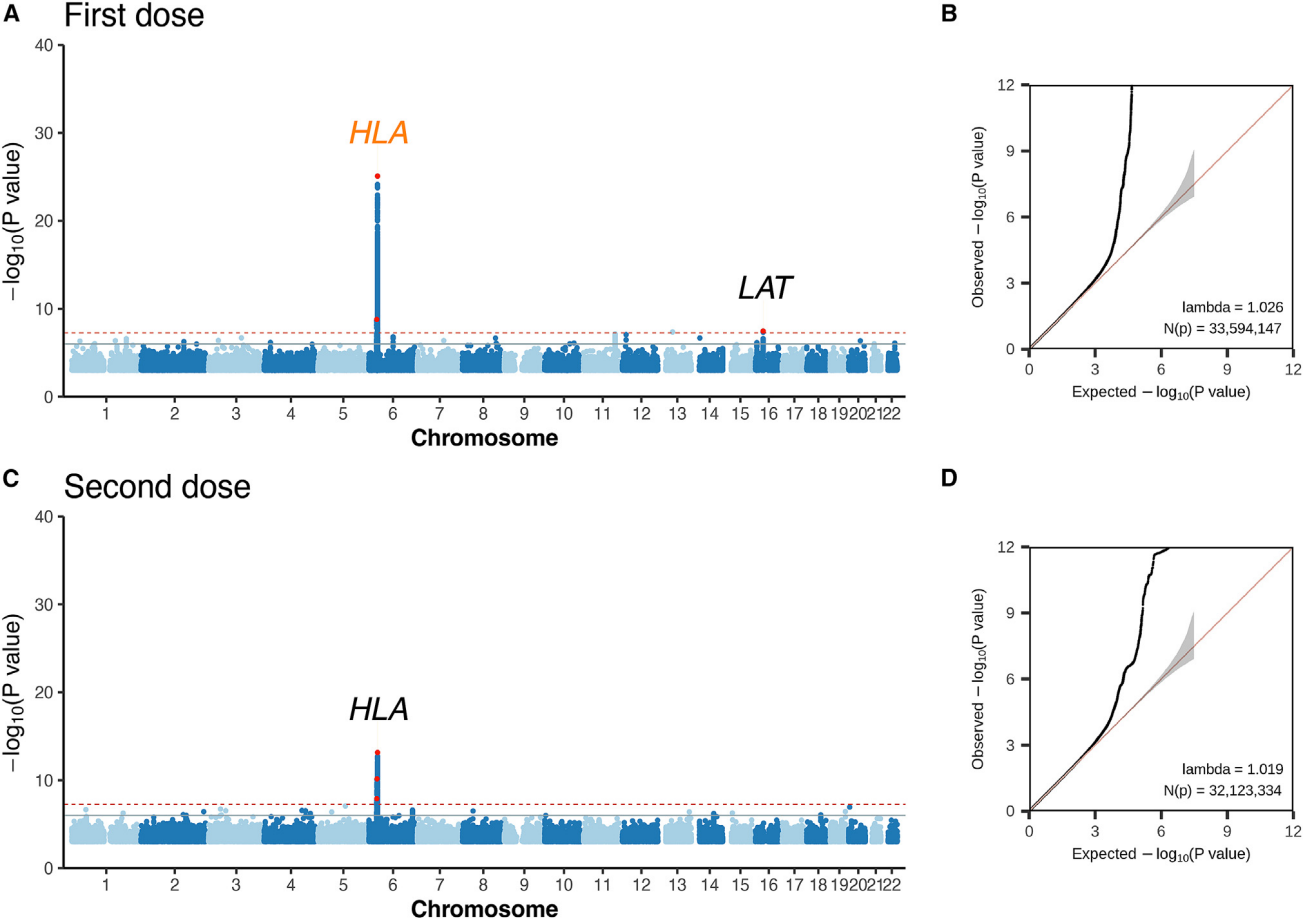
Genome-wide association results of host response to COVID-19 vaccine in combined cohorts (A) Manhattan plot of serostatus following the first-dose vaccination. The red dots represent the lead SNPs with a MAF > 5% within 500 kbp blocks. The labeled symbols correspond to the nearest genes or regions of the lead SNPs. If the lead SNP is replicated in the other two cohorts, the font color is orange; otherwise, it is black. The red horizontal line corresponds to the genome-wide significance threshold p value ≤ 5e−8, and the blue horizontal line represents the genome-wide suggestive significance threshold p value ≤ 1e−6. (B) Q-Q plot of serostatus following the first-dose vaccination. The gray shade represents the 95% confidence interval of the expected distribution. (C) Manhattan plot of serostatus following the second-dose vaccination. The gene label font color and horizontal lines are consistent with (A). (D) Q-Q plot of serostatus following the second-dose vaccination.

Generalized mixed models implemented in SAIGE 1.09 (May 17, 2022) were used for common-variant genome-wide association analysis.^23^ The two phenotypes (serostatus for first-dose and second-dose vaccination) were analyzed in the three cohorts separately for imputed dosages and analyses were adjusted for age, sex, the first twenty principal components (which were determined by Tracy-Widom test and consistent with previous study^19^), and the antibody test kit brands as an additional covariate for the combined cohort. For every analysis, genetic relationship matrix (GRM) was constructed using 50,000 hard-called genotyping markers. Only variants with a minor allele count (MAC) above 10 were included in the GWAS analysis. Statistical significance was set to the traditional level of p < 5e−8 while suggestive significance was set to p < 1e−6. The variant with minimum p value in 500 kbp was considered to be the lead SNP. SNPs with p value less than 5e−8 in the combined cohort and below the Boferroni-corrected p value (0.05/4 = 0.0125 for the first dose and 0.05/3 = 0.016 for the second dose) in both the replication and discovery cohorts were considered to be the replicated variants (Table 2) and were marked in orange in the Manhattan plot (Figure 2).

**Table 2:** Conditionally independent and replicated SNP/allele/amino acid within the HLA region

**Trait**	**Round**^a^	**SNP (gene)/allele/amino acid**	**BP**	**Eff/Ref/df**^b^	**Combined**			**Discovery**			**Replication**		
					**FRQ**^c^	**p_con_**^d^	**OR/n**^h^	**FRQ**	**p_0_**^e^	**OR/n**^h^	**FRQ**	**p_0_**	**OR/n**^h^
**HLA_SNP**													

first dose	0	rs2858331 (*XXbac-BPG254F23.7*)	32,681,277	G/A	0.378	9.31e−26	1.19	0.378	2.80e−11	1.15	0.378	2.59e−16	1.22
first dose	1	rs10947290 (*HLA-DRB9*)	32,446,269	A/G	0.071	5.06e−16	0.8	0.073	9.44e−10	0.81	0.073	8.08e−19	0.69
first dose	2	rs35986240 (*HLA-DQB1*)	32,630,991	A/G	0.033	5.29e−10	1.31	0.033	9.64e−6	1.3	0.033	1.34e−5	1.32

**HLA_allele**													

first dose	0	*HLA-DRB1^∗^13:02*	32,546,547	p/a	0.040	2.34e−16	0.75	0.041	4.51e−8	0.78	0.039	5.61e−11	0.7
first dose	1	*HLA-DQA1^∗^01:01*	32,605,183	p/a	0.141	4.92e−10	1.11	0.140	5.07e−6	1.13	0.143	3.20e−9	1.2
first dose	2	*HLA-DPB1^∗^04:01*	33,043,703	p/a	0.461	1.64e−8	0.92	0.460	7.43e−4	0.94	0.461	4.03e−5	0.91
first dose	3	*HLA-DQB1^∗^02:01*	32,627,241	p/a	0.152	9.54e−6	0.88	0.153	1.64e−3	0.9	0.151	3.59e−5	0.86

**HLA_AA_binary**^f^													

first dose	0	AA_DRB1_71_exon2_Arg	32,551,949	p/a	0.446	1.88e−25	1.18	0.446	2.24e−10	1.14	0.447	9.16e−19	1.23
first dose	1	AA_DQB1_130_exon3_Gln	32,629,933	p/a	0.039	6.19e−9	0.81	0.039	3.72e−8	0.78	0.038	3.79e−11	0.7
first dose	2	AA_DPB1_215_exon4_Ile	33,053,582	p/a	0.314	1.25e−8	1.1	0.314	1.73e−5	1.09	0.316	5.58e−8	1.14

**HLA_AA_omnibus**^g^													

first dose	0	AA_DRB1_71	32,551,949	3	–	5.48e−23	54,066	–	7.71e−10	34,632	–	2.28e−17	20,078
first dose	1	AA_DQB1_130	32,629,933	2	–	1.04e−13	54,066	–	3.22e−7	34,632	–	1.58e−11	20,078
first dose	2	AA_DPB1_205	33,053,552	2	–	8.21e−5	54,066	–	6.63e−5	34,632	–	1.16e−7	20,078
first dose	3	AA_DQB1_87	32,632,571	3	–	9.46e−5	54,066	–	5.82e−6	34,632	–	2.05e−4	20,078

Amino acid residues are presented in the form AA_<gene>_<position>_<exon>_<amino acid residue>.

a The round number of conditional analyses.

b Effect and reference state for SNP, allele, and binary; degrees of freedom for the omnibus test. For SNP, there are alternative and reference sites, and for amino acid and allele, "p" means present, "a" means absent. For the omnibus test, df is equal to the number of residues at this position − 1.

c The allele frequency of effect site in cohort.

d Conditional p value for the corresponding round in the conditional analysis.

e p value without conditional analysis.

f Amino acid association calculated by binary test.

g Calculate amino acid position association using omnibus test.

h OR is shown for SNP, allele, and binary; n (sample size of study cohort) is shown for omnibus test.

### Fine-mapping in HLA region

To determine independent association signals for phenotypes showing a genome-wide significance in the HLA region and to investigate which amino acid position plays a role in the host response to a COVID-19 vaccine, we utilized a forward stepwise regression strategy separately for four classes of markers in HLA region: SNP, HLA allele binary markers, amino acid residue binary markers, and amino acid positions. PLINK 2.0^21^ (19 May 2022 version) was used for the conditional association analysis of the first three binary markers, and the likelihood ratio test, omnibus test, was used for the association analysis of amino acid positions using HATK.^24^ In condition analysis, selected variants’ allelic dosage were added as fixed covariates in the regression model which could directly account for linkage disequilibrium (LD).

A total of 182,789 SNPs located from 28,510,120 to 33,480,577 on chromosome 6 (human genome region MHC assembly GRCh37) were included in HLA region analysis, two phenotypes in each of the three cohorts. Covariates were the same as for the GWAS. The HLA^∗^IMP:02 algorithm with a multi-population reference panel was used for the imputation of classical HLA alleles for UKBB participants at 4-digit resolution.^19^^,^^25^ Detailed information about the HLA imputation can be seen in UKBB (https://biobank.ndph.ox.ac.uk/showcase/label.cgi?id=100035).

HATK was used to convert HLA allele names to the most up-to-date naming conventions and to generate binary markers representing the presence/absence of each allele or amino acid residue with IMGT/HLA database 3.47.0 (15 March 2022)^26^^,^^27^ for eight classical HLA genes: *HLA-A*, *HLA-B*, and *HLA-C* (class I) and *HLA-DPA1*, *HLA-DPB1*, *HLA-DQA1*, *HLA-DQB1*, and *HLA-DRB1* (class II). We started by performing unconditional analysis in three cohorts. Markers were considered replicated if they were significant in the combined cohort and also significant in the other two cohorts (combined cohort threshold = 0.05/total number of tests; replication threshold = 0.05/number of significant markers in the combined cohort). To be specific, for SNPs, combined cohort threshold was equal to 5e−8 and replication threshold was equal to 2.02e−5 (0.05/2,481); for HLA allele binary markers, combined cohort threshold was equal to 2.58e−4 (0.05/194) and replication threshold was equal to 2.5e−3 (0.05/20); for amino acid residue binary markers, combined cohort threshold was equal to 5.52e−5 (0.05/905) and replication threshold was equal to 3.16e−4 (0.05/158); and for amino acid positions, combined cohort threshold was equal to 1.31e−4 (0.05/382) and replication threshold was equal to 6.02e−4 (0.05/83). Replicated markers were selected to perform the following forward stepwise iteration in the combined cohort in which markers with the lowest p value will be recognized and set as covariates until no significant marker left satisfying a p value less than the first significant threshold.

### Sensitivity analysis

We assessed the robustness of the primary findings by introducing adjustments to the phenotype definition and the association analysis model. This evaluation encompassed the following key modifications.

(1) Inclusion of the time between vaccination and antibody testing as covariates within the association model
(2) Imposition of an age restriction, limiting the analysis to individuals aged 40–64 years
(3) Application of a specific time frame constraint for antibody testing, permitting analysis only for seronegative individuals within 7–210 days after the second vaccine
(4) Similar to the third adjustment, a restricted time frame between the second vaccine dose and antibody testing (7–210 days) was applied for seronegative individuals, with this time frame also introduced as a covariate in the association analysis
(5) Formation of subset comprising individuals with information on vaccine type, utilizing data sourced from GP prescription records (category 996 in UKBB); the type of vaccine received was integrated as a covariate in the association analysis

### Cross-ancestry association analysis and meta-analysis

We defined three non-European ancestry populations within UKBB by integrating genetic ancestry and self-reported race/ethnicity using the Harmonized Ancestry and Race/Ethnicity (HARE) approach.^28^ The genetic ancestry of unrelated individuals was inferred using the first 30 principal components recommended by the software, with the default parameters “t1 = 20, t2 = 40” of HARE for classification. The methods employed for analyzing each ancestry cohort regarding serostatus after first-dose or second-dose vaccination were consistent with those applied to the European ancestry group. In brief, we conducted the cross-ancestry meta genome-wide association analysis using METAL with default parameters,^29^ and we performed cross-ancestry meta association analysis of the four classes of markers in HLA region—SNP, HLA allele binary markers, amino acid residue binary markers, and amino acid positions—using PLINK2^21^ and HATK,^24^ following the same established protocol as mentioned above.

### 3D protein structure modeling and electrostatic potential calculations

The protein sequences of the target classical HLA alleles were downloaded from the IMGT/HLA database.^26^^,^^27^ We used 3PDO in SWISS-MODEL^30^ and Protein Data Bank^31^ as the modeling template for HLA-DR protein. In order to obtain a protein closer to the complete structure, the target HLA-DR molecules was modeled in SWISS-Model with the alpha chain of 3PDO as hetero targets. Essential hydrogens and missing sidechain atoms were added to structures using PDB: 2PQR (http://www.poissonboltzmann.org/) and the protonated molecule was then used to compute the electrostatic potential. Electrostatic potentials around the resulting 3D structures were generated using Adaptive Poisson-Boltzmann Solver (APBS) plugin within PyMOL software (v.2.3.2).^32^

### TWAS and spTWAS analysis

UTMOST^33^ (https://github.com/Joker-Jerome/UTMOST) was run as a single-tissue association test for whole blood tissue from GTEx project^34^^,^^35^ and three immune cell types, namely CD14^+^ monocytes, CD16^+^ neutrophils, and naive CD4^+^ T cell from BLUEPRINT consortium^36^ (single_tissue_association_test.py). The test used the GWAS summary files of two phenotypes, namely serostatus for first-dose vaccination and second-dose vaccination as input. Other necessary command parameters were used by default. TWAS and spTWAS significance threshold for single tissue analysis is p value < 7.56e−7 (0.05/number of tests in 4 tissues or cell types for two phenotypes in combined cohorts = 0.05/66160 = 7.56e−7. We also define replication of significant associations: according to the above threshold, there are 30 significant gene-tissue/cell pairs in combined cohorts, and they were considered replicated if they achieve p < 0.05/30 = 1.67e−3 in the other two cohorts.

## Results

### Study design and demographic characteristics of the study cohorts

We utilized data from two studies from UK Biobank: the 200K self-test antibody study and the 60K Coronavirus infection study, to define two phenotypes according to the criteria outlined in the methods section (Figure 1). Briefly, we primarily examined serostatus in 78,876 individuals after the first dose of vaccination within 20–60 days and in 77,232 individuals after the second dose of vaccination within 0–300 days using a self-test kit. Individuals with no detectable S-protein IgG level were classified as IgG seronegative (cases), while individuals with detectable S-protein IgG and no detectable N-protein antibody were classified as IgG seropositive (controls). Individuals with positive N-protein antibodies were excluded to ensure that seropositivity reflected the immune response to the vaccine and not an exogenous infection of SARS-CoV-2. Detailed information on the inclusion and exclusion criteria for the case/control groups is presented in Table S1.

According to the S antibody detection kit, each phenotype was divided into three cohorts: the discovery cohort (AbC-19TM Rapid Test), the replication cohort (Fortress Fast test kit), and the combined cohort (discovery cohort + replication cohort). To mitigate the influence of population stratification and cryptic relatedness, we conducted the primary analysis excluding samples that were not of European ancestry or were related within three degrees, while the cross-ancestry analysis results were conducted for comparative purposes and was detailed below. Analyzing the serostatus of the cohorts, we found that 56.4%–76.0% of participates did not have detectable antibodies after the first vaccination, 31.9%–49.8% of participates still did not produce detectable antibodies after the second vaccination (Table S2), and 88.6% (19,737/22,272) of the seronegative individuals were present from 0 to 50 days after the second vaccination (Figure S1). Except for the absence of a significant correlation between country of birth and serostatus for first-dose vaccination, sex, age, and country of birth were all correlated with serostatus for both first-dose and second-dose vaccination (p < 0.05) (Table S2).

Subtle significant difference in age, Townsend deprivation, sex, country of birth, and vaccine type were observed between the discovery and replication cohorts (p < 0.05) (Table 1). Principal component analysis (PCA) indicates consistent scatterplot distribution and geographic structure of samples among the three cohorts (Figures S2–S4). We further examined the statistical differences in genotype frequency of HLA alleles among subpopulations from different birthplace countries. For the first-dose vaccination combined cohort, 14 alleles significantly differed across subpopulations from different birthplace countries (p_Bonferroni_ threshold = 2.16e−4), including 9 alleles in HLA class I and 5 alleles in class II (*HLA-DRB1^∗^03:01*, *HLA-DRB1^∗^15:01*, *HLA-DQB1^∗^02:01*, *HLA-DQB1^∗^06:02*, and *HLA-DPB1^∗^04:02*). In the second-dose vaccination combined cohort, 9 alleles exhibited significant differences, including 6 alleles in HLA class I and 3 alleles in HLA class II (*HLA-DRB1^∗^03:01*, *HLA-DRB1^∗^11:01*, and *HLA-DQB1^∗^02:01*) (Table S3). We controlled for substructure among Europeans by using principal components as covariates. The Tracy-Widom test showed that for all cohorts, the top 8, 11, 15, 16, and 16 PCs were significant (p < 0.05) (Table S4). Following the Tracy-Widom test results and a previous study,^19^ we chose PCs 1–20 to account for the potential effects of population structure.

### HLA class II variants play the major role in genetically determining the IgG serostatus after COVID-19 vaccination

Regarding serostatus after the first vaccine, the estimated heritability for IgG serostatus in the combined cohort was 3.13% (SE = 0.89%) according to LDSC.^37^ After adjusting for age, sex, and the first twenty principal components, we identified genetic loci associated with three independent common lead SNPs (MAF > 5%, p < 5e−8) in the combined cohort with a linear mixed model implemented in SAIGE (Figure 2A; Table S5; see subjects and methods). Specifically, the two lead SNPs, rs2858331-G (OR[95%CI] = 1.19[1.15,1.22], p = 8.28e−26) and rs2523409-G (OR[95%CI] = 1.1[1.07,1.13], p = 1.60e−9) in the HLA region (chr6:28,477,797–33,448,354, GRCh37), were consistently found across two kits used to measure serostatus. While rs2523409-G has not been reported in the GWAS catalog,^38^ rs2858331-G, located between *HLA-DQB1* and *HLA-DQA2*, has been associated with serum IgE levels and immunoglobulin A vasculitis.^39^

In addition to the HLA region, a genetic locus near *LAT* on chromosome 16, tagged by a synonymous variant, was also significantly associated with the risk of seronegativity after the first vaccine dose (lead SNP rs1131543-A, OR[95%CI] = 1.08[1.05,1.12], p_combined_ = 3.43e−8, p_discovery_ = 5.61e−9, p_replication_ = 0.04) (Figures S5 and S6). LAT (linker for activation of T cells) is phosphorylated by ZAP-70/Syk protein tyrosine kinases following activation of the T cell antigen receptor (TCR) signal transduction pathway,^40^ and our study suggested a potential association with serostatus after the first dose of vaccination. Analysis of expression quantitive trait loci (eQTL) in blood tissue using data from the eQTLGen Consortium^41^ revealed that the rs1131543-A allele regulatse *TUFM* (p = 7.95e−59) and *SPNS1* (p = 7.72e−56). *TUFM* plays a role in the regulation of autophagy and innate immunity and is associated with infectious disease and SARS-CoV-2 infection.^42^ *SPNS1* has been identified as a host factor that facilitates SARS-CoV-2 virus entry into lung tissue, possibly through the supply of lysophospholipids that enhance membrane fusion.^43^ These findings suggest a potential relationship between the host IgG response to COVID-19 vaccination and the susceptibility and severity of COVID-19. Locuszoom plot (https://genome.sph.umich.edu/wiki/LocusZoom) comparing the association signals provides further evidence for this hypothesis (Figure S7).

In the discovery cohort of the first vaccine analysis, we also observed a cluster of significant signals on chromosome 14 (p_discovery_ < 5e−8) (Table S6; Figure S6). However, this signal was not replicated in the replication cohort (p_replication_ = 0.04). The lead SNP rs8013923-C (p_discovery_ = 1.21e−8, OR[95%CI] = 1.12 [1.08,1.17]) is located upstream of the protein-coding gene *IGHV1-66* (Figure S5). There is a high level of linkage disequilibrium between rs8013923-C and variants present in *IGHV1-66* according to Ldpair (R2 = 1.0, D’ = 1.0, p < 1e−4).^44^ Several studies have demonstrated that antibodies composed of the protein encoded by *IGHV3-6* have a disproportionately high utilization rate in individuals with COVID-19, referred to as public antibodies.^45^^,^^46^^,^^47^

Regarding serostatus after the second-dose vaccination, the estimated heritability for the combined cohort was 2.98% (SE = 1.03%) based on LDSC.^37^ We identified three independent genetic loci tagged by lead SNPs rs66656759-T, rs3094106-C, and rs3128911-C in the HLA region. However, these signals were not replicated between the discovery and the replication cohorts (Figure 2B; Table S5). Future analyses are required to validate and understand these unreplicated findings in our study. For the subsequent discussion, we focused on fine-mapping and interpreting the results that were consistent between the two independent cohorts.

### Fine-mapping of independent associations of three replicated SNPs with IgG serostatus after the first dose of COVID-19 vaccine in the HLA class II region

**Figure 3:**
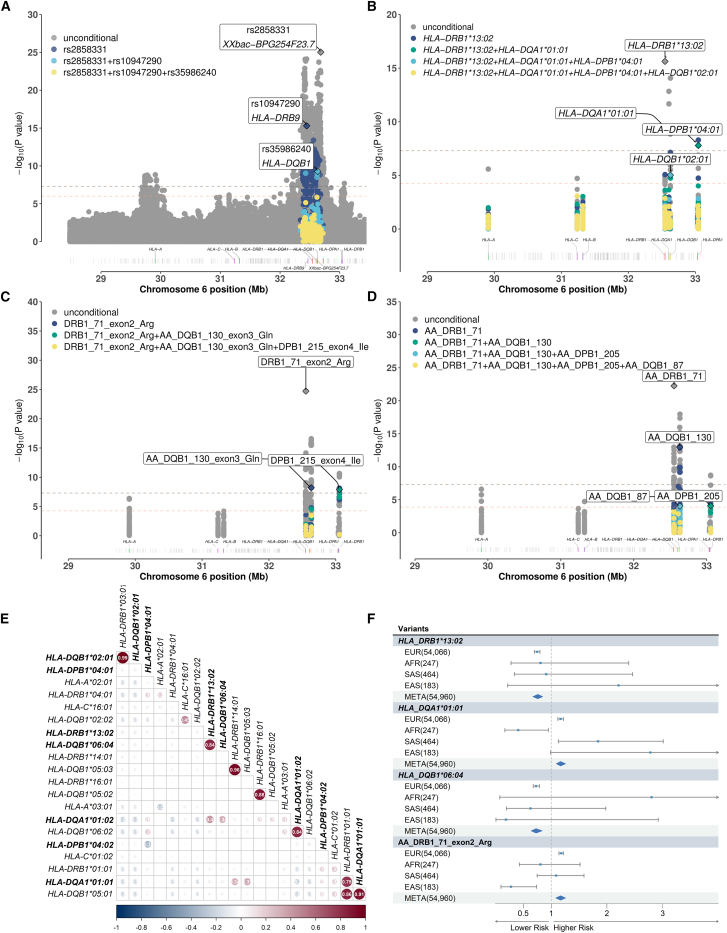
Conditionally associated SNP/allele/amino acid in HLA region for serostatus after the first-dose vaccination (A) Conditional association plots of SNP in HLA region. Genome regions with only gray dots indicate associations that were not replicated in other cohorts. Therefore, conditional analyses were not performed for these associations. The labeled associations represent the lead SNP and its nearest gene in each round of conditional analysis. The legend on the upper left indicates the covariate included in each round of conditional analysis. The upper horizontal dashed line represents the genome-wide significance threshold p value ≤ 5e−8, and the horizontal dashed line below represents the genome-wide suggestive threshold p value ≤ 1e−6. (B) Conditional association plots of alleles of eight classical HLA genes. The horizontal dashed line below represents the Bonferroni-corrected threshold 2.58e−4 for HLA regional association analysis, while the other elements remain the same as in (A). (C) Conditional association plots by binary test of amino acids for eight classical HLA genes. The labeled associations represent the lead amino acid association in each round of conditional analysis, provided in the form of gene, position on the protein, exon, and amino acid residue abbreviation. The horizontal dashed line below represents the Bonferroni-corrected threshold of 5.52e−5 for amino acid association analysis in the HLA region, with the remaining elements consistent with (A). Single letter is a shortened version of the three-letter amino acid code: I, Ile; Q, Gln; R, Arg. (D) Conditional association plots by omnibus test of amino acids for eight classical HLA genes. The horizontal dashed line below represents the Bonferroni-corrected threshold of 1.31e−4. (E) Pairwise correlation plot between the alleles that are significant in at least one cohort. The bold font indicates significant and replicated alleles. The value in the cell is Pearson’s correlation coefficient. (F) The significant and replicated alleles that are also significant in cross-ancestry meta analysis. The numbers inside parentheses indicate the sample size. The blue dots and the middle of the diamonds represent the point estimates of the odds ratios. The line segments and the left and right boundaries of the diamonds represent the 95% confidence intervals of the odds ratios.

The above GWAS analysis suggests a central role of HLA class II region in regulating the host response to COVID-19 vaccination. Given the intricate linkage disequilibrium pattern within the HLA, we performed conditional analysis to fine-map specific variants within this region. Initially, we identified significant polymorphisms in the HLA region, which were consistently replicated in the three cohorts (p < 5e−8 in combined cohort, p < 2.02e−5 in discovery and replication cohorts) (Figure 3A; Table S7). Iterative analyses were conducted by conditioning on the lead SNP and adding the lead SNP as a covariate, until all loci were no longer significant (p > 5e−8). Ultimately, we identified three independent and replicated association SNPs (Figure 3A; Table 2): rs2858331-G in round 1 (no conditioning), rs10947290-A in round 2 (conditioning on rs2858331-G), and rs35986240-A in round 3 (conditioning on rs2858331-G and rs10947290-A). All three SNPs were located in the HLA class II region.

For rs2858331-G in round 1, annotation results indicate that its location upstream of the lincRNA *XXbac-BPG254F23.7* (Ensembl: ENSG00000232080) situated between *HLA-DQB1* and *HLA-DQA2*. This SNP has previously been associated with various phenotypes such as IgE levels and immunoglobulin A vasculitis in the GWAS catalog.^38^ For rs10947290-A in round 2, the annotation reveals its location upstream of *HLA-DRB9*, a pseudogene between *HLA-DRB1* and *HLA-DRA*. Although this SNP has not been reported in the GWAS catalog,^38^ the OpenGWAS database^48^ shows its significant associations with several rheumatoid arthritis conditions (OpenGWAS ID = ebi-a-GCST90013534, bbj-a-74, ieu-a-833, ebi-a-GCST002318, bbj-a-73, ieu-a-832, bbj-a-151), with p values ranging from 9.96e−27 to 3.27e−310. In addition, OpenGWAS indicates that this SNP is significantly associated with the expression of multiple genes, including *HLA-DRB1*, *HLA-DRB5*, *HLA-DRB6*, *HLA-DQA1*, *HLA-DQA2*, *HLA-DQB1*, *HLA-DQB1-AS1*, *HLA-DQB2*, and *SKIV2L*. For rs35986240-A in round 3, the annotation results show its location within an intron of *HLA-DQB1*. While not reported in the GWAS catalog,^38^ OpenGWAS highlights its significant associations with rheumatoid arthritis (beta = −0.63, SE = 0.06, p = 4.38e−23, OPENGWAS ID = ebi-a-GCST90013534), type 1 diabetes (beta = −0.77, SE = 0.09, p = 5.51e−18, OPENGWAS ID = finn-b-E4_DM1NOCOMP), and systemic lupus erythematosus (beta = 0.24, SE = 0.04, p = 5.23e−9, OPENGWAS ID = ebi-a-GCST90011866).

In summary, these three SNPs are situated within the HLA class II region, and their proximity to specific genes suggests a potential association between DR-related and DQ-related proteins and the serostatus for the initial dose of COVID-19 vaccines. Furthermore, PheWAS analysis in OpenGWAS indicates that these SNPs impact the expression genes in the HLA class II region. Consequently, we investigate allele and amino acid residue associations within the major HLA class II genes in the following.

### Independent associations of *HLA-DRB1*^∗^13:02, *HLA-DQA1*^∗^01:01, *HLA-DPB1*^∗^04:01, and *HLA-DQB1*^∗^02:01 with serostatus after the first COVID-19 vaccination

HLA class II antigens play a crucial role in recognizing and presenting antigens derived from extracellular sources.^49^ To further investigate the relationship between different types of HLA class II coding genes and the host’s response to COVID-19 vaccine, we performed an association analysis on alleles of eight classical HLA genes: *HLA-A*, *HLA-B*, and *HLA-C* (class I) and *HLA-DPA1*, *HLA-DPB1*, *HLA-DQA1*, *HLA-DQB1*, and *HLA-DRB1* (class II) at a resolution of 4 fields. Conditional analysis, adjusting for age, sex and the first twenty principal components, were conducted on significant and replicated alleles using an iterative approach similar to the SNP analysis mentioned earlier.

Regarding serostatus after the first-dose vaccination, we identified four independent alleles that show significant and consistent replication across the three cohorts (Figure 3B; Table 2). These alleles were located within the HLA class II region, aligning with the SNP results. Specifically, in the first round of conditional analysis, *HLA-DRB1^∗^13:02* consistently demonstrated a strong protective effect against IgG seronegativity following the first COVID-19 vaccination (OR[95%CI] = 0.75[0.7,0.8], p_condition_ = 2.34e−16). In a longitudinal cohort study involving 1,364 healthcare workers (HCWs) from five hospitals in the UK, the presence of *HLA-DRB1^∗^13:02* was associated with a 6- to 7-fold increased risk of symptomatic COVID-19 cases among seropositive (S-protein or N-protein) individuals (n = 272),^15^ suggesting a potential link of the allele to severe COVID-19. In the second round, *HLA_DQA1^∗^01:01* was associated with a higher risk of non-response to the first dose of the vaccine (OR[95%CI] = 1.11[1.10,1.19], p_condition_ = 4.92e−10). In third round, *HLA_DPB1^∗^04:01* demonstrated a significant protective effect (0.92[0.9,0.95], p_condition_ = 1.64e−8). In the fourth round, *HLA_DQB1^∗^02:01* also displayed a protective effect (0.88[0.83,0.93], p_condition_ = 9.54e−6). Importantly, previous studies have indicated that *DQB1^∗^02:01* was significantly associated with the antibody response to the inactivated Japanese encephalitis vaccine (p < 0.001, OR[95%CI] = 0.364, 95% CI: 0.221–0.600),^50^ suggesting that it may be a host factor associated with a broad reduction in immune response to vaccines. What’s more, we plotted a pairwise correlation matrix between alleles that were significantly associated with serostatus after first-dose vaccination in at least one cohort using European genetic data (Figure 3E). This plot showed the correlation between alleles and were consistent with the conditionally independent alleles selected by the conditional analysis. Based on it and the results of the conditional analysis, the seven significant and replicated alleles in the article can be classified into four groups: (1) *HLA-DRB1^∗^13:02*, *HLA-DQB1^∗^06:04*, and *HLA-DQA1^∗^01:02*; (2) *HLA-DQA1^∗^01:01*; (3) *HLA-DPB1^∗^04:01* and *HLA-DPB1^∗^04:02*; and (4) *HLA-DQB1^∗^02:01*. This suggests a potential haplotype.

### The amino acid residue at position 71 on DRβ1 and its pocket electrical properties are associated with serostatus following the first COVID-19 vaccination

To obtain a deeper understanding of the mechanism underlying the associations of HLA genetic changes and the IgG vaccine responses, we continue to conduct a regression analysis of the amino acid residues of HLA proteins and their associated phenotypes. In addition, we conducted surface electrostatic interaction analysis to understand the function of the replicated and independently associated amino acids.

For serostatus following the first dose of vaccines, three independent and significant amino acids were identified and replicated: arginine (Arg) at position 71 of HLA-DRβ1 (pocket 4 in the peptide binding groove), glutamine (Gln) at position 130 of HLA-DQβ1, and isoleucine (Ile) at position 215 of HLA-DPβ1 (Figure 3C). In the first round (without any conditions), HLA-DRβ1-Arg71 was positively associated with seronegativity (OR[95%CI] = 1.18[1.22,1.14], p = 1.88e−25). In the second round, after conditioning on HLA-DRβ1-Arg71, an independent signal was observed at HLA-DQβ1-Gln130 (OR[95%CI] = 0.81[0.75,0.87], p_condition_ = 6.19e−9). In the third round, after conditioning on HLA-DRβ1-Arg71 and HLA-DQβ1-Gln130, we observed an independent signal at HLA-DPβ1-Ile215 (OR[95%CI] = 1.1[1.06,1.13], p_condition_ = 1.25e−8). Finally, when conditioning on these three amino acids, all other amino acids became insignificant (Figure 3C). All three associations were successfully replicated (Table 2).

**Figure 4:**
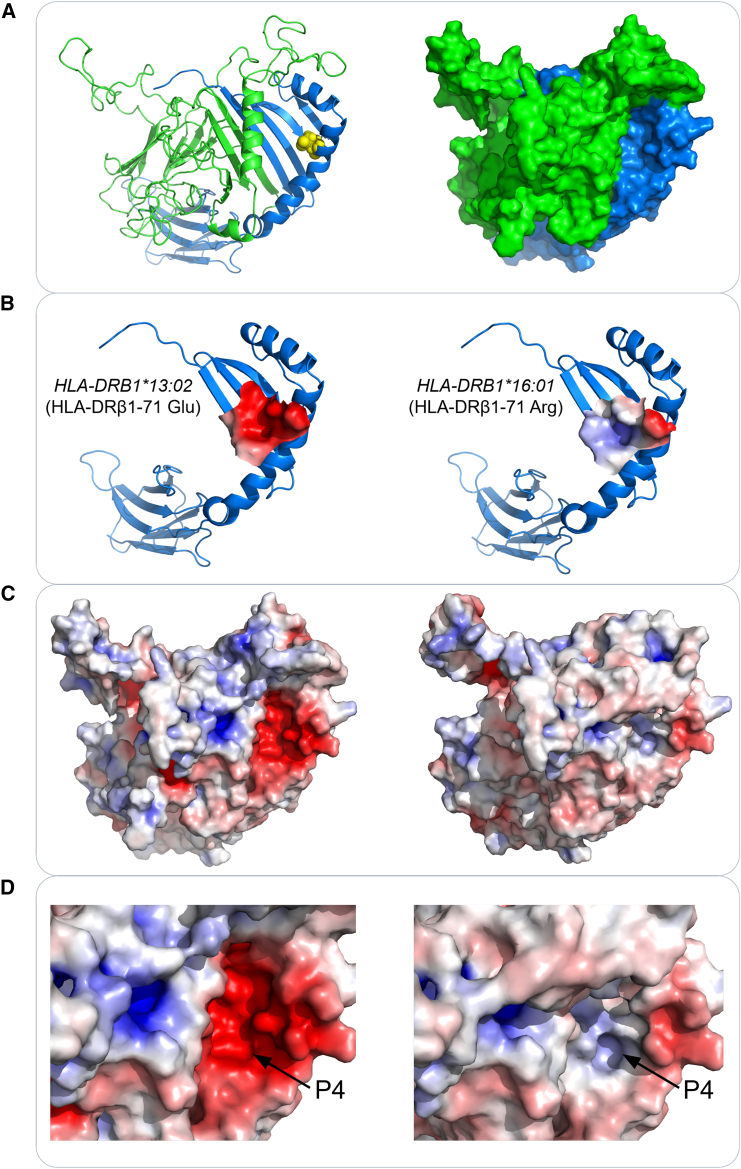
Structure and surface electrostatic potential of HLA-DRβ1 molecule (A) Cartoon structure and surface structure of the complex composed of HLA-DRβ1 (represented by HLA-DRβ1^∗^13:02) and the reference α chain molecule. The reference α chain is colored green and the β chain is colored blue. Glu/Arg 71 residue is located at the P4 pocket of HLA-DQβ1. (B) Cartoon structure of single chain and surface electrostatic potential of P4 pocket for HLA-DRβ1^∗^13:02 and HLA-DRβ1^∗^16:01, respectively. The P4 pocket of HLA-DRβ1^∗^13:02 exhibits a negatively charged pocket, while HLA-DRβ1^∗^16:01 possesses a positively charged P4 pocket. (C and D) The surface electrostatic potential of HLA-DRβ1^∗^13:02 and HLA-DRβ1^∗^16:01 in complex with the reference α chain molecule. These figures provide a closer representation of the actual and comprehensive surface electrostatic potential at the P4 pocket. Regions with negatively charged potentials (less than 5 kt/e) are colored red, those with positively charged potentials (greater than 5 kt/e) are colored blue, and neutrally charged potentials (0 kT/e) are colored white.

Among the three replicated independent association amino acids, HLA-DRβ1-Arg71 is located in pocket 4 of the peptide binding groove. We speculate that this amino acid may influence the electrostatic potential of pocket 4, thereby altering its binding ability to antigenic peptides and subsequently influencing antibody production. Therefore, in the combined cohort, we ranked the p values from lowest to highest and excluded *HLA-DRB1* alleles that were linked to other significant alleles. Consequently, the top two significant *HLA-DRB1* alleles identified were *HLA-DRB1^∗^13:02* and *HLA-DRB1^∗^16:01* (Table S8). We compared the electrostatic potential energy in HLA-DRβ1 pocket 4, as shown in Figure 4: *HLA-DRB1:13:02* (OR[95%CI] = 0.75[0.7,0.8], p = 2.34e−16) with glutamic acid (Glu) at position 71 (OR[95%CI] = 0.92[0.88,0.96], p = 7.06e−16) (Tables 2 and S9) carried a negative charge in pocket 4, while *HLA-DRβ1^∗^16:01* (OR[95%CI] = 1.53[1.25,1.88], p = 3.11e−5) with arginine (Arg) at position 71 (OR[95%CI] = 1.18[1.14,1.22], p = 1.88e−25) (Tables 2 and S8) carried a positive charge in pocket 4. These observations suggest a link between the direction of electrostatic potential energy in pocket 4 of HLA-DRβ1 and the IgG serostatus for the first-dose vaccination. Specifically, a negative charge promotes the interaction between HLA-DRβ1 and the vaccine antigen, while a positive charge has the opposite effect.

The other two independent amino acids are not located in the peptide binding groove and their electrostatic potential energy cannot be analyzed. The positions of the replicated and independent amino acids identified by the omnibus test align with the binary test, indicating the reliability of the results (Figure 3D; Tables 2 and S10).

### Consistent estimation of HLA genetic effects in sensitivity analyses

To assess potential influence of various factors on our primary findings, such as vaccine type, age criteria, and the duration between vaccination and antibody test, we conducted a series of sensitivity analyses with adjustments made to phenotype definitions and covariates (subjects and methods). These sensitivity analyses consistently reaffirmed the reliability of our primary results (Tables S11–S19). For a GWAS for the two phenotypes, the cluster of significant association signals in the HLA region of chromosome 6 persisted even when subjected to the abovementioned adjustments (Figure S8). For the association analysis of SNPs/alleles/amino acids within the HLA region, we compared the conditionally independent and replicated variants from the primary analysis in the sensitivity analyses. As a result, the variants that were conditionally independent in the first two rounds retained their significance and the reported associations were successfully replicated in these sensitivity analyses. Notably, there were no significant changes in the genetic effect value or direction trends, especially in the case of the most significantly associated allele, *HLA-DRB1^∗^13:02,* and the amino acid variant of HLA-DRβ1-Arg71, which constituted the primary focus of our investigation (Table S19).

### Cross-ancestry genetic associations with IgG response

To understand the genetic basis of IgG vaccine responses in non-European populations, we extended our analysis to three non-European ancestry populations across UKBB, including 7,284 individuals of African ancestry (AFR), 8,850 individuals of South Asian ancestry (SAS), and 1,713 individuals of East Asian ancestry (EAS). We established six cohorts with sample sizes ranging from 113 to 541 with accompanying IgG measurements (Figure S9) for serostatus after the first-dose or second-dose vaccination (subjects and methods). However, due to the relatively limited sample sizes, we did not identify any additional genome-wide significant variants unique to a specific non-European population.

Regarding the HLA alleles and amino acids, we observed significant associations related to serostatus after the first vaccine dose among the EAS group. Specifically, HLA-DQβ1-Pro55 (OR = 0.22[0.11,0.45], p = 2.82e−5) and *HLA-DQB1^∗^03:03* (OR = 0.17[0.07,0.41], p = 8.55e−5) demonstrated significant associations that withstood the stringent Bonferroni correction criteria (Table S20). We did not identify any additional significantly associated alleles or amino acids within the other non-European ancestral groups. After meta-analysis, for serostatus after the first vaccine dose, we identified three significantly associated alleles: *HLA-DRB1^∗^13:02* (identified as the leading allele in conditional analysis round 0 in analysis of the European population), *HLA-DQA1^∗^01:01* (the leading allele in conditional analysis round 1 in analysis of the European population), and *HLA-DQB1^∗^06:04* (which exhibited linkage disequilibrium with *HLA-DRB1^∗^13:02*) (Figures 3E and 3F). None of the amino acids reached significance levels below the Bonferroni-corrected p value threshold of 5.16e−5, while HLA-DRβ1-Arg71 was the most significant (p = 8.67e−5) and approached the threshold (Figure S9; Table S20). As for serostatus after the second vaccine, we identified five significantly associated alleles and no amino acid associations (Figure S9; Table S21).

Furthermore, we embarked on exploring the relationship between the *HLA-DRB1^∗^13:02* allele, which exhibited the most significant association with seropositivity following the first vaccine (Table 2), and vaccine efficacy. We used the Allele Frequency Net Database (http://allelefrequencies.net/) to estimate the allele frequency of *HLA-DRB1^∗^13:02* in three ancestry groups (African, European, and Asian ancestry group) through sample size weighted averaging. We compared these population-specific allele frequencies with efficacy statistics of BNT162b2 and CHAdOx1 (the two COVID-19 vaccine mainly used in our study) from two large-scale multi-ethnic studies.^51^^,^^52^ We found a consistent pattern wherein the frequency of *HLA-DRB1^∗^13:02* progressively decreased across the African, European, and Asian ancestry groups, registering values of 7.34%, 4.85%, and 2.40%, respectively (Table S22). Remarkably, vaccine efficacy for these two different vaccines exhibited a parallel decrease across the three ancestry groups, standing at rates of 91.9% (African), 91.3% (European), and 87.6% (Asian) for BNT162b2 and 91.8% (African) and 73.1% (European) for ChAdOx1 (no data available for Asian). This alignment in changes in allele frequency suggests that there is a potentially essential role for inherent genetics in determining vaccine efficacy. However, further studies are warranted, incorporating more individual data from diverse ancestral groups and controlling for potential confounding effects rising from socioeconomic disparities among different ethnic groups to quantify this effect accurately.

### Cell-type-specific effect of HLA class II genes on COVID-19 vaccine response

In the investigation of SNPs in the HLA region, we found that certain significant and independent SNPs were linked to the expression of multiple HLA class II genes. One of these notable SNPs is the replicated independent lead SNP rs10947290-A. To explore the connection between gene expression and outcomes, we conducted transcriptome-wide association analysis (TWAS) and splicing transcriptome association analysis (spTWAS) in whole blood tissue and three immune cell types using the UTMOST algorithm.^33^

**Figure 5:**
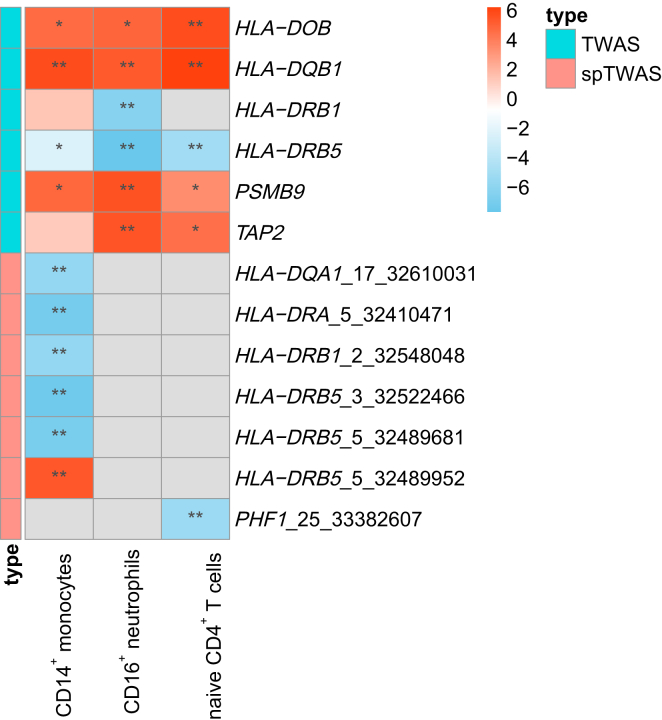
Distribution of *Z* scores across gene-cell pairs by TWAS and spTWAS for serostatus following the first-dose vaccination The gene-cell pair indicated by ^∗∗^ represents a significant p value in the combined cohort, surpassing the Bonferroni-corrected threshold and being replicated in other cohorts. The gene-cell pair indicated by ^∗^ represents a p value < 0.05 in the combined cohort. Blue or red cells without ^∗^ indicate p values > 0.05, while gray cells indicate no test results were generated. The labels on the right of spTWAS heatmap are the phenotype IDs in BLUEPRINT dataset (https://ftp.ebi.ac.uk/pub/databases/blueprint/blueprint_Epivar/qtl_as/QTL_RESULTS/).

In the TWAS analysis, we investigated serostatus following the first-dose vaccination and observed nine significant and replicated gene-cell-phenotype pairs, including *HLA-DOB*, *HLA-DQB1*, *HLA-DRB1*, *HLA-DRB5*, *PSMB9*, and *TAP2* (p < 7.56e−7). Notably, the replicated gene expression associations were primarily observed in neutrophils (5 out of 9), with none detected in whole blood (Figure 5; Tables S23 and S24). Particularly, the predicted expression of *DQB1* in the three cell types exhibited a significant association with seronegativity, and this association was replicated (5.16 < *Z* < 6.49, p = 8.35e−11 in CD4^+^ T cells, p = 1.12e−8 in CD14^+^ monocytes, and p = 2.52e−7 in CD16^+^ neutrophils). Furthermore, the predicted expression of *HLA-DRB1* in neutrophils demonstrated a significant protective association with seronegativity and was replicated (*Z* = −6.50, p = 7.55e−11). Additionally, the predicted expression of *PSMB9* (encoding a proteasome subunit) and *TAP2* (encoding an ATP-binding cassette transporter) in CD16^+^ neutrophils both displayed a significant association with seronegativity and was replicated (*Z* = 5.50, p = 3.79e−8). Interestingly, previous transcriptome studies have shown significantly higher expression of *PSMB9* and *TAP2* in individuals with COVID-19 compared to healthy control subjects, suggesting their potential involvement in excessive inflammation in individuals with COVID-19.^53^^,^^54^

Using spTWAS, we identified seven significant and replicated gene-cell-phenotype pairs, including *HLA-DQA1*, *HLA-DRA*, *HLA-DRB1*, *HLA-DRB5*, and *PHF1* (p < 7.5e−7). These replicated gene expression associations were predominantly observed in CD14^+^ monocytes (6 out of 7) (Figure 5; Tables S23 and S24). Specifically, the predicted relative abundance of alternatively spliced transcripts of *HLA-DRB1* in CD14^+^ monocytes exhibited a significant negative association with seronegativity and was replicated (*Z* < −3, p = 1.33e−8). Additionally, the predicted relative abundance of alternatively spliced transcript of *HLA-DQA1* in CD14^+^ monocytes exhibited a significant association with seropositive subjects and was replicated (*Z* < −3, p = 5.82e−9). Finally, the predicted relative abundance of three alternatively spliced transcripts of *HLA-DQA1* displayed a significant association with serostatus and was replicated (Figure 5; Tables S23 and S24).

## Discussion

Our study conducted a powerful and comprehensive genome-wide association study to investigate the influence of host genetic factors on the IgG response to COVID-19 vaccine in individuals without prior infection. In the study design, we incorporated two pivotal advantages to mitigate potential technical artifacts in the GWAS. Firstly, we differentiated the response to the COVID-19 vaccine from the response to SARS-CoV-2 infection by excluding individuals with a seropositive result for N-protein antibodies in their blood plasma, thus ensuring the dissection of genuine genetic effects associated with COVID-19 vaccine response. Secondly, we conducted replication studies to assess genetic effects on serostatus using two different experimental kits. In addition, the substantial sample size (n_first-dose_ = 54,066, n_second-dose_ = 46,232) provided significantly increased statistical power, enabling a more precise and robust analysis of genetic associations compared to smaller studies (n_first-dose_ = 78).^15^

In the genome-wide association analysis, we identified a significant role of the HLA class II genes in affecting the serostatus after the initial dose of the COVID-19 vaccination. Through conditional analyses on the alleles of eight classical genes within the HLA region at a 4-field resolution, we discovered and replicated four significant and independent alleles, namely *HLA-DRB1^∗^13:02*, *HLA-DQA1^∗^01:01*, *HLA-DPB1^∗^04:01*, and *HLA-DQB1^∗^02:01*, accounting for the variability in IgG response to the vaccine. Furthermore, our analysis of amino acid residues identified three independent and replicated residues, including the 71Glu/Arg substitution in HLA-DRβ1. This specific substitution alters the electrostatic potential of pocket 4 and exhibited a significant association with seronegativity following the initial COVID-19 vaccination. Moreover, by leveraging TWAS and spTWAS, we identified nine significant and replicated genes (including five HLA class II genes) with predicted expression levels associated with the serostatus after the first dose of the COVID-19 vaccine in at least one immune cell type. This finding implies that both the protein sequence and expression levels of HLA class II genes contribute to the serostatus resulting from the initial COVID-19 vaccine dose.

We postulate that the amino acid residue polymorphism influences the binding affinity of the antigen peptide of the COVID-19 vaccine to the T cell receptor (TCR) via alteration of the electrostatic potential of the fourth pocket (P4) of HLA-DRβ1, thereby impacting the efficiency of the immune response. Specifically, our results indicate that the replicated and conditionally independent allele *HLA-DRB1^∗^13:02*, carrying a basic amino acid Glu at position 71 and an overall negative charge on P4, is significantly associated with seropositivity after the first dose of the vaccine. Conversely, the presence of Arg at position 71 and an overall positive charge on P4 (associated with allele *HLA-DRB1^∗^16:01* and is not linked to other significant alleles), is associated with seronegativity after the first dose of the vaccine. A recent GWAS conducted in the UK involving 1,076 participants reported two genetic associations, *HLA-DQB1^∗^06* and HLA-DRβ1-71Glu/Arg, with IgG levels after the first dose of vaccination.^16^ However, the relationship between *HLA-DQB1^∗^06* and HLA-DRβ1-71Glu/Arg is unclear. In our study, we observed that the *HLA-DQB1^∗^06:04* allele and the most significant allele *HLA-DRB1^∗^13:02* were in linkage disequilibrium, and that the 71 position of DRβ1 for *HLA-DRB1^∗^13:02* was significantly correlated with the serostatus after the first dose of vaccination. These findings support that the *HLA-DRB1^∗^13:02* allele was more likely a potential causal allele, which may affect the eletrostatic potential of P4 through amino acid 71Glu and thereby enhance antigen binding.

Previous research has demonstrated that the peptide binding site of HLA class II molecules is formed by the N-terminal domains of the alpha and beta chains, with both chains contributing to approximately half of the floor as well as one of the two long helices that form the peptide binding site.^55^^,^^56^ Peptide binding to the antigen peptide occurs through two mechanisms: (1) a sequence-independent mode involving the formation of hydrogen bonds between the backbone of the peptide and conserved residues of the MHC class II binding site and (2) a sequence-dependent interaction mode in which peptide side chains occupy specific pockets of the binding site.^55^^,^^56^ Findings from the current study support that the second mechanism, which involves interactions between the amino acid residue at position 71 of HLA-DRβ1 and the antigen peptide side chains, plays an important role in the host immune response to the COVID-19 vaccine. Moreover, research has shown that β71 is in a position that binds to the TCR,^56^^,^^57^ which may affect the subsequent immune process. It has been reported that amino acid polymorphisms or electrical properties of DRβ P4 are associated with immune-related diseases such as rheumatoid arthritis (RA), pemphigus vulgaris (PV), and multiple sclerosis (MS),^58^^,^^59^^,^^60^ further highlighting the important role of this region in immunity. For example, in RA, the positively charged P4 pocket in RA-associated subtypes *HLA-DRB1^∗^04:01*, *HLA-DRB1^∗^04:04*, and *HLA-DRB1^∗^01:01* (due to a basic residue, either lysine or arginine, at position β71) is implicated in disease susceptibility, while *HLA-DRB1^∗^04:02* subtypes that do not confer RA susceptibility carry a negative charge at positions β70 and β71 in the P4 pocket.^58^ Peptide binding studies have shown that the β71 polymorphism is particularly important for determining binding specificity in RA-associated subtypes.^61^

Studying the genetic factors influencing the host response to COVID-19 vaccine provides valuable insights into factors that influence COVID-19 susceptibility and severity. Although the COVID-19 vaccine does not contain live virus, its design is based on the S protein of the virus, making the host’s immune response to vaccine antigens a part of the immune process against the virus. It is worth noting that while the vaccine response study conducted here defines affected individuals and control subjects based on serostatus, defining COVID-19 susceptibility and severity typically relies on population-derived data, which may introduce confounding factors related to the HLA regions.^62^ Through association analysis, we found that certain variants were associated with both the response to COVID-19 vaccine and the COVID-19 susceptibility and severity. These variants point to the HLA class II region, where the encoded proteins primarily participate in the recognition and antigen presentation of foreign antigens. This aligns with previous research on the genetic contribution of seroreactivity to a few other viruses, which has also identified the HLA as a key determinant of antibody response.^63^ A recent large-scale WGS association study identified a significant association between the lead SNP rs9271609, located upstream of *HLA-DQA1* and *HLA-DRB1* in the HLA class II region, and COVID-19 severity.^64^ The *HLA-DRB1^∗^13:02* was also associated with 6- to 7-fold increased risk of case definition symptomatic COVID-19 in seropositive (S-protein or N-protein) populations (n = 272) from a longitudinal cohort study (n = 1,364) of 5 hospital UK healthcare workers (HCWs).^15^ These results support a shared genetic basis between the vaccine response and COVID-19 outcomes within the HLA class II region.

In addition to the HLA genes, we found that an association signal around the *LAT* (16q13) locus (lead SNP rs1131543-A, OR[95%CI] = 1.08[1.05,1.12]). The immune response to COVID-19 vaccine is a T cell-dependent antibody response, where T cells are activated by antigen-presenting cells (APCs) and subsequently proliferate and differentiate into specific CD4^+^ T cells. This process is essential for promoting the proliferation and differentiation of corresponding B cells into plasma cells. The activation of T cells occurs through the binding of T cell receptors (TCRs) to peptide-MHC complexes of APCs. This binding triggers a signaling cascade involving the phosphorylation of immunoreceptor tyrosine-based activation motifs in the CD3 chains by LCK. Subsequently, the protein tyrosine kinase ZAP70 is recruited and activated, leading to the phosphorylation of the transmembrane adaptor protein LAT. This event results in the formation of the LAT signalosome, which recruits various adaptors and effector molecules to regulate cellular biochemical processes. If there is a problem with LAT on the signal transduction chain, it will affect the proliferation, migration, cytokine production, and effector function of CD4^+^ T cells, and B cells cannot proliferate and differentiate into specific plasma cells.^40^ Notably, a proteomic blood profiling study reported a significant association between *LAT* expression and COVID-19 susceptibility.^65^ Furthermore, we observed that this lead SNP rs1131543-A significantly regulated the expression of two genes: *TUFM* and *SPNS1*. These genes are involved in infectious disease pathways and have been implicated as host factors facilitating SARS virus entry into lung tissue,^42^^,^^43^ further supporting that the relationship between the host response to COVID-19 vaccine and susceptibility and severity of COVID-19.

Finally, we found that the influence of HLA alleles on IgG responses varies depending on the cell type involved. Specifically, we observed a significant association between IgG responses and predicted gene expression in CD16^+^ neutrophil cells and naive CD4^+^ T cells. Of note, the predicted expression of *PSMB9* was significantly associated with seronegativity after the first dose of the COVID-19 vaccine, as revealed by TWAS and replication. Additionally, a separate transcription study of proteasome subunits revealed a significant association between the expression of *PSMB9* in blood and susceptibility to COVID-19 infection.^53^ These findings highlight the importance of further investigating individuals who are seronegative after COVID-19 vaccination due to underlying immunogenetic factors. Such individuals may be more susceptible to infection or at risk of developing a severe outcome.

We acknowledge several limitations in our study. First, our study predominantly involves individuals of European origin from UKBB. While cross-ancestry analyses have been conducted, the findings for non-European populations are constrained by relatively smaller sample sizes, necessitating further expansion. Second, our definition of the phenotype classified affected individuals as seronegative within specific time frames after vaccine doses: 20 to 60 days after receiving the first vaccine dose and within 300 days of receiving the second dose. However, we were unable to distinguish individuals with an inadequate response to the vaccine (“primary vaccine failures”) from those unable to maintain serological immunity over time (“secondary vaccine failures”). Notably, in the sensitivity analyses adding the duration between vaccination and serology test and adjusting time thresholds, we obtained consistent results, confirming the stability of our findings. Third, the S antibody detection technology used in our study, based on colloidal gold technology, has a higher detection limit compared to laboratory methods, which could lead to false negatives. However, these false-negative results also reflect the low levels of vaccine-induced immunity in the case group. By reporting consistent results from the two independent cohorts assayed with different serologic tests, we eliminated possible false positive signals derived from this issue. Fourth, for the second dose phenotype, we were unable to replicate the association loci, necessitating further replication in independent cohorts. Fifth, our primary analysis did not incorporate vaccine type as a covariate due to missing information in nearly 50% of the samples across cohorts. However, sensitivity analyses that included vaccine type as a covariate in the remaining samples consistently indicated that vaccine types had little effect on the results. This may be because the primary vaccine types received by UKBB participants targeted the S protein. Finally, the exclusion of vaccinated but naturally infected individuals from our study cohorts may introduce bias into the genetic distribution, potentially resulting in a lower frequency of shared genetic variation related to SARS-CoV-2 infection and response to COVID-19 vaccine and thus limit our ability to detect such associations. To address this concern, we conducted genetic association studies between those who receive the vaccination without positive N antibodies (uninfected population) and those who receive the vaccination but were excluded in the primary analysis because of positive N antibodies (naturally infected population) for the first and second vaccination phenotypes, respectively. Results for both cohorts revealed few significant loci, especially for chromosome 6 (Figure S10). Furthermore, the results of association analysis for alleles/amino acids only display a significantly different allele *HLA-C^∗^07:02* (p = 1.86e−4) for the first-dose vaccination after Bonferroni correction (Tables S25 and S26). These findings indicate that the genetic frequency of the cohort established in this study did not have obvious genetic bias, and the results can be well generalized to a broader population.

In conclusion, this study unravels HLA class II genes as the major genetic determinants influencing the IgG antibody response to COVID-19 vaccination and explored the underlying mechanisms. These findings provide insights into the biological mechanism underlying individual variation in response to COVID-19 vaccine and may have broader implications for understanding humoral responses for other vaccines. Our findings also highlight the importance of considering the influence of constitutive genetics when designing vaccination strategies for optimal protection across diverse populations. Given the persistent impact of COVID-19 and the inevitability of future pandemics, the results derived from this study lay the groundwork for prevention and control of infectious disease.

## Data and code availability

The GWAS summary statistics are available in the GWAS Catalog (accession numbers GCST90295945 - GCST90295946) with URLs: https://ftp.ebi.ac.uk/pub/databases/gwas/summary_statistics/GCST90295001-GCST90296000/GCST90295945/ and https://ftp.ebi.ac.uk/pub/databases/gwas/summary_statistics/GCST90295001-GCST90296000/GCST90295946/.

## Supplemental information

Data S1. Tables S1–S26

Document S2. Article plus supplemental information
