# Supplementary material for: Genetic determinants of IgG antibody response to COVID-19 vaccination
Source: Am J Hum Genet. 2024 Jan 4;111(1):181–99. doi: 10.1016/j.ajhg.2023.12.005 (PMC10806743; doi:10.1016/j.ajhg.2023.12.005)
Supplement: Document S1. Figures S1–S10 [file mmc1.pdf]

The American Journal of Human Genetics, Volume *111*

Supplemental information

Genetic determinants of IgG antibody response to COVID-19 vaccination

Shengzhe Bian, Xinxin Guo, Xilai Yang, Yuandan Wei, Zijing Yang, Shiyao Cheng, Jiaqi Yan, Yongkun Chen, Guo-Bo Chen, Xiangjun Du, Stephen S. Francis, Yuelong Shu, and Siyang Liu

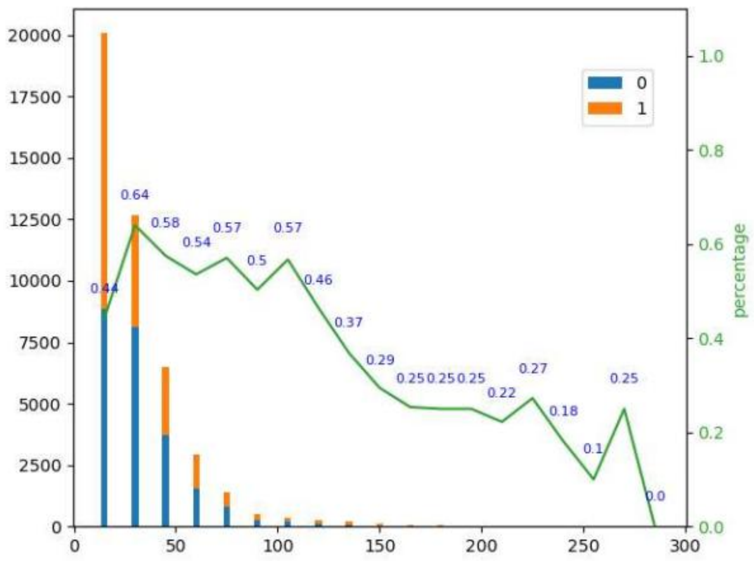

Figure S1-Distribution of the time interval between the administration of the second dose vaccine and serology test in the combined cohort covering the timeframe of 0 to 300 days. The x-axis denotes the duration between the second vaccine dose and the serology test in combined cohort, with a histogram bin width of 15 days. The left y-axis represents the number of individuals, while the right y-axis represents the rate of antibody positivity. The line graph overlaid on the bars presents the antibody positivity rate in each bar. The “0” in the legend represents the control group and “1” represents the case group.

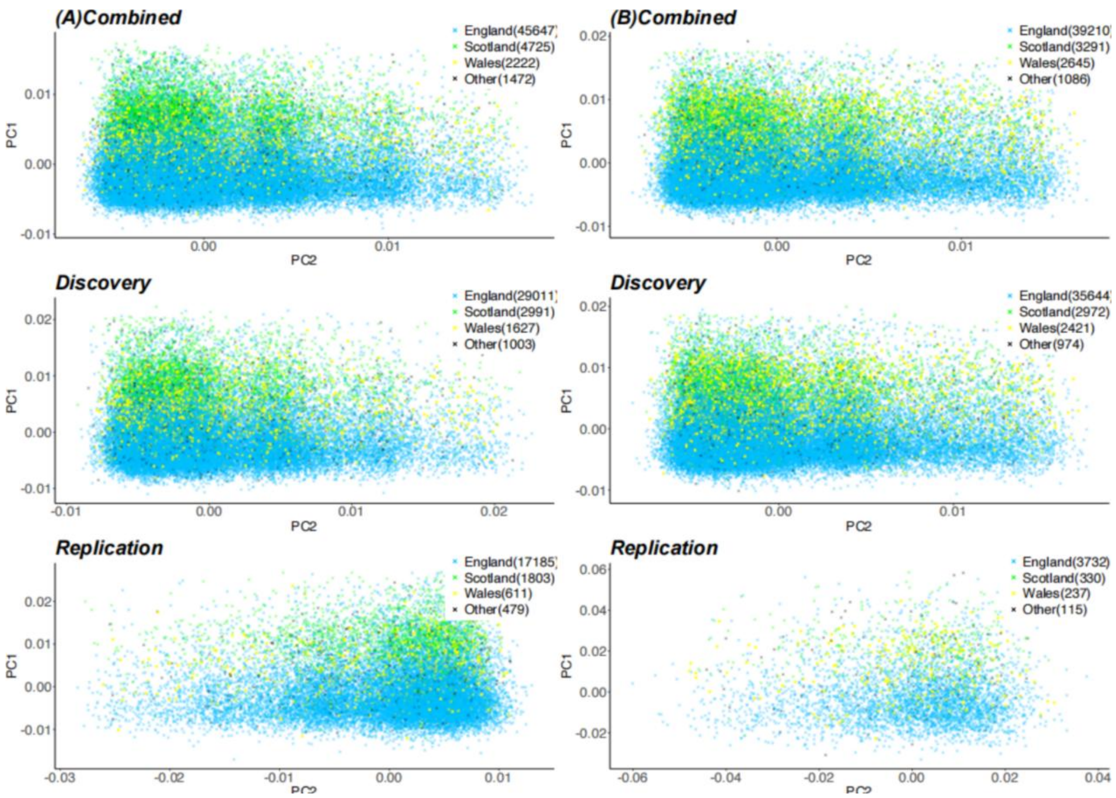

Figure S2-Scatter plot of PC1-2 of every cohort. Left: first dose vaccine. Right: second dose vaccine

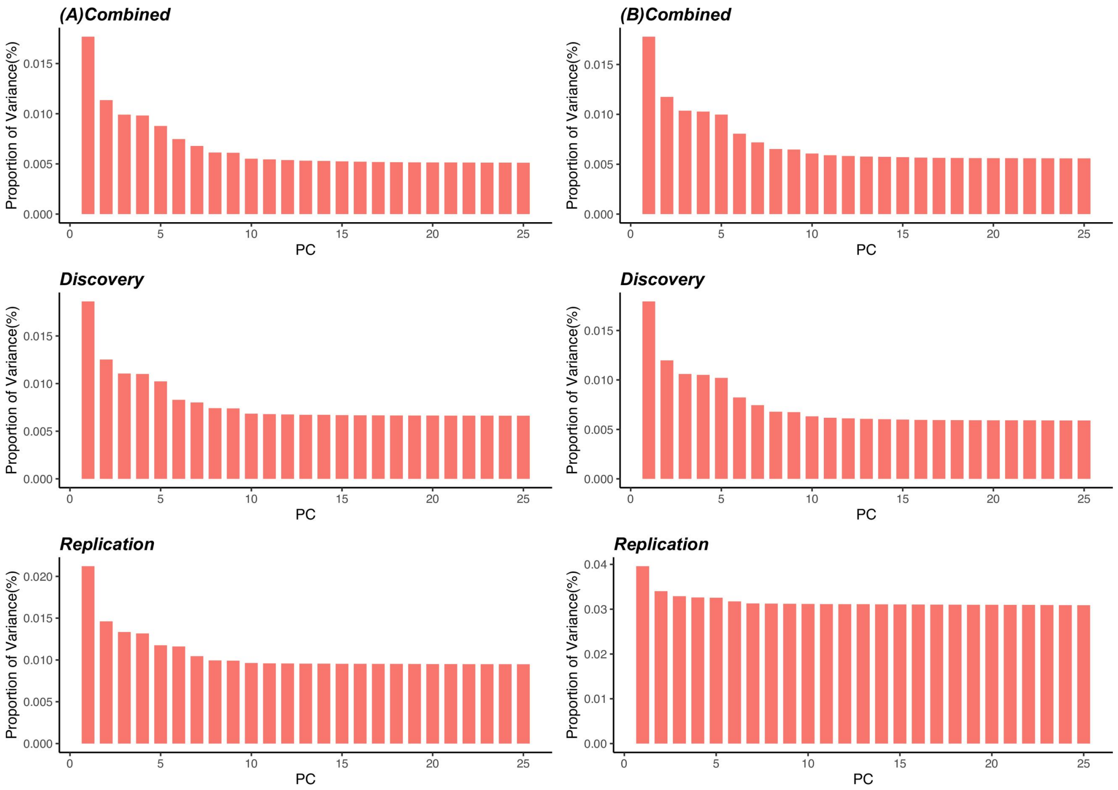

Figure S3-The proportion of variance for PC1-25 of every cohort. Left: first dose vaccine. Right:second dose vaccine

(A)

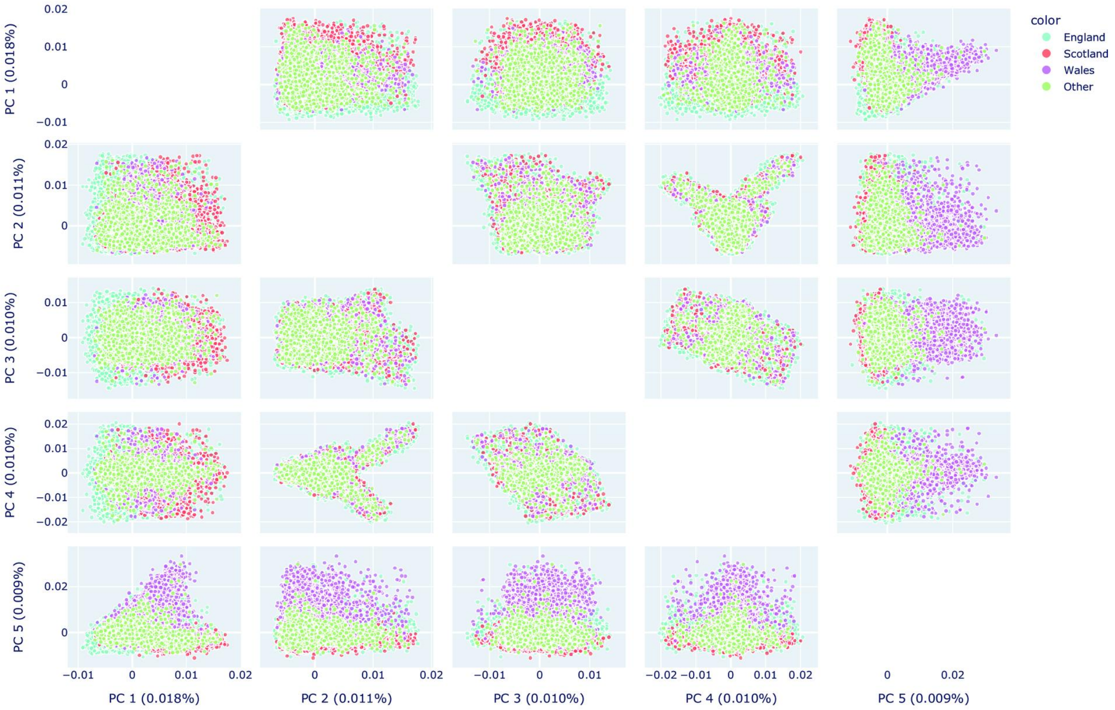

(B)

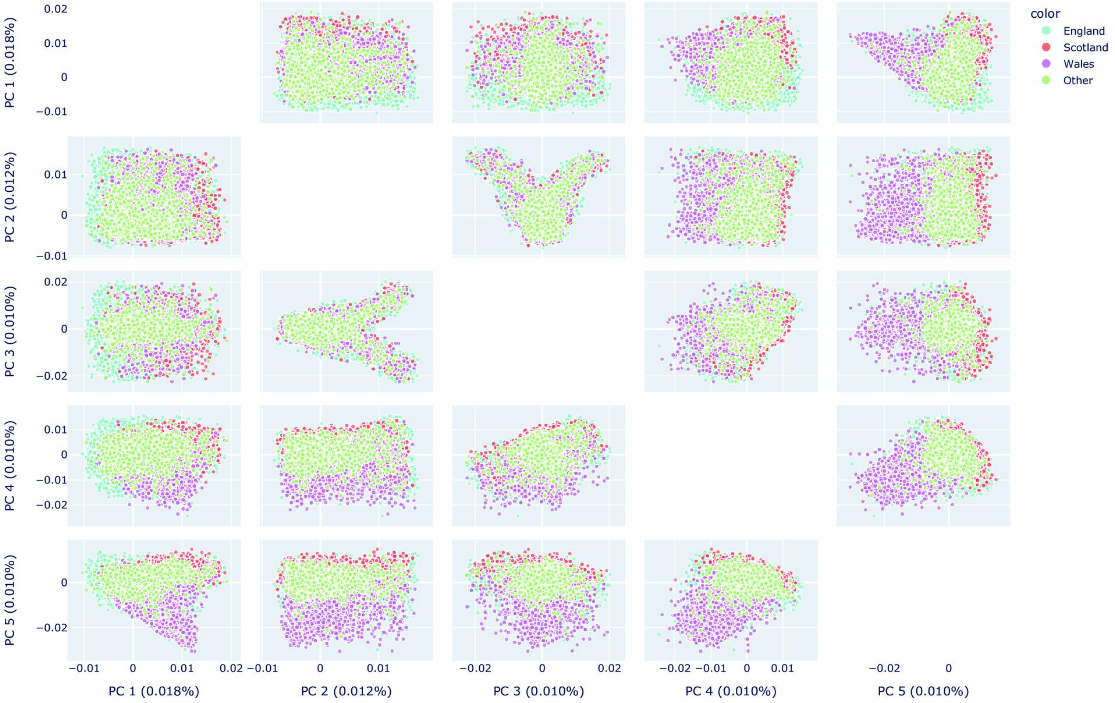

Figure S4- Scatter plots of PCs 1-5 for the combined cohort. (A) Scatter plot for the combined cohort following the first-dose vaccination in the combined cohort. **(B)** Scatter plot for the combined cohort following the second-dose vaccination.

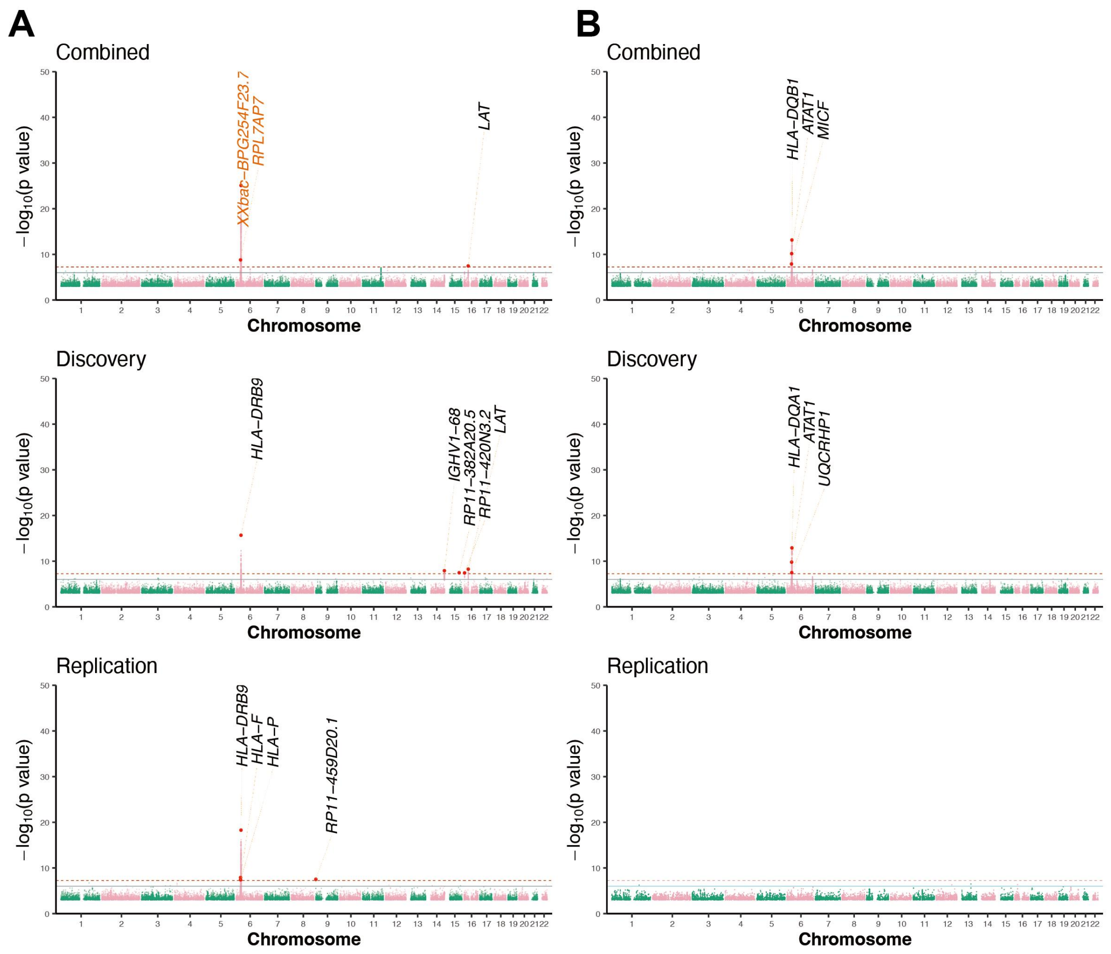

Figure S5-The manhattan plot of every study cohort. (A) GWAS for serostatus for the first-dose vaccination. **(B)** GWAS for serostatus for the second-dose vaccination.

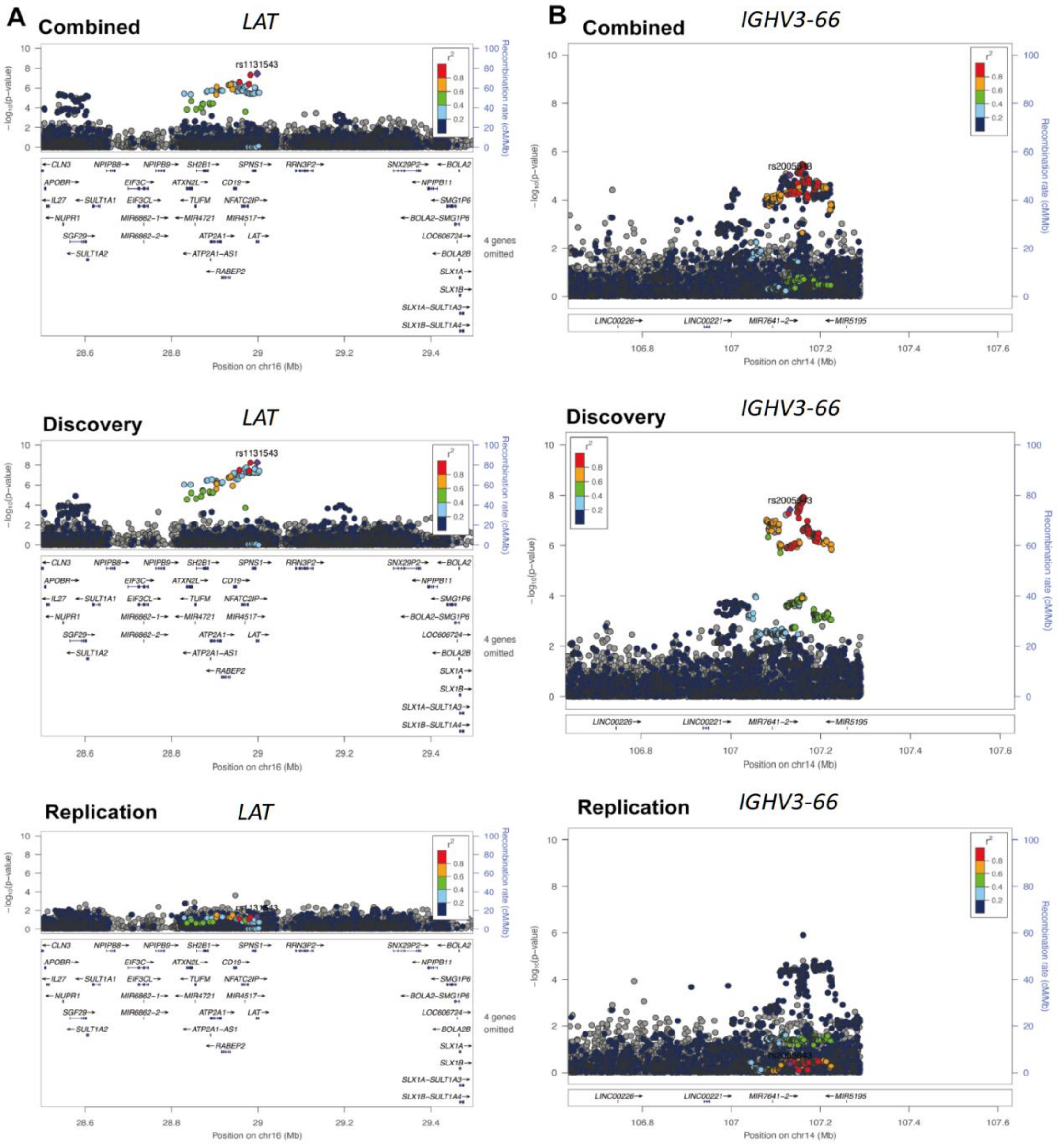

Figure S6-The locuszoom plot of *LAT* in chromosome 16 and *IGHV3-66* in chromosome 14 of serostatus for the first-dose vaccination GWAS.

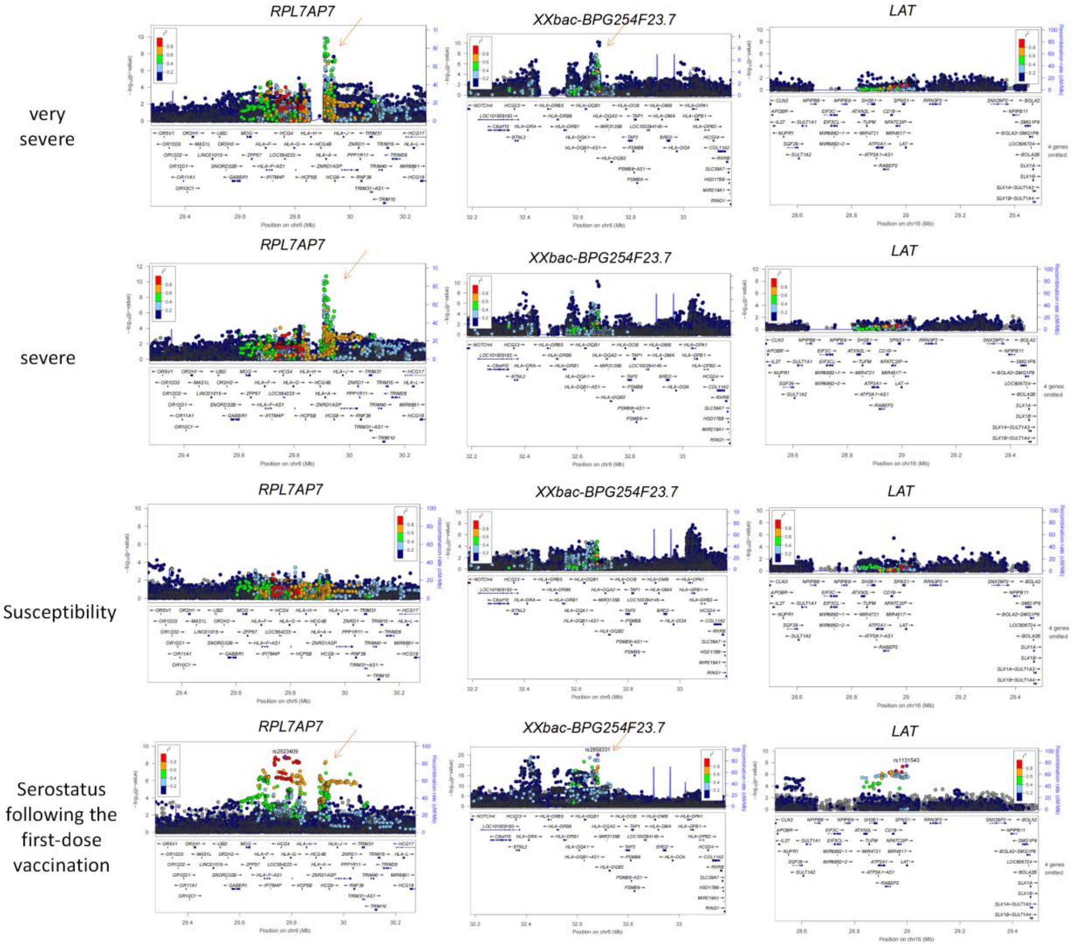

Figure S7-The locuszoom plot comparing the genome-wide association signals for vaccine IgG response and COVID-19 susceptibility and severity. GWAS summary statistics for very severe COVID-19, severe COVID-19, and susceptibility were obtained from the COVID-19 host genetics initiative with following links.

(https://storage.googleapis.com/covid19-hg-public/freeze_7/results/20220403/main/sumstats/COVID19_HGI_A2_ALL_leave_23andme_20220403_GRCh37.tsv.gz
https://storage.googleapis.com/covid19-hg-public/freeze_7/results/20220403/main/sumstats/COVID19_HGI_B2_ALL_leave_23andme_20220403_GRCh37.tsv.gz
https://storage.googleapis.com/covid19-hg-public/freeze_7/results/20220403/main/sumstats/COVID19_HGI_C2_ALL_leave_23andme_20220403_GRCh37.tsv.gz)

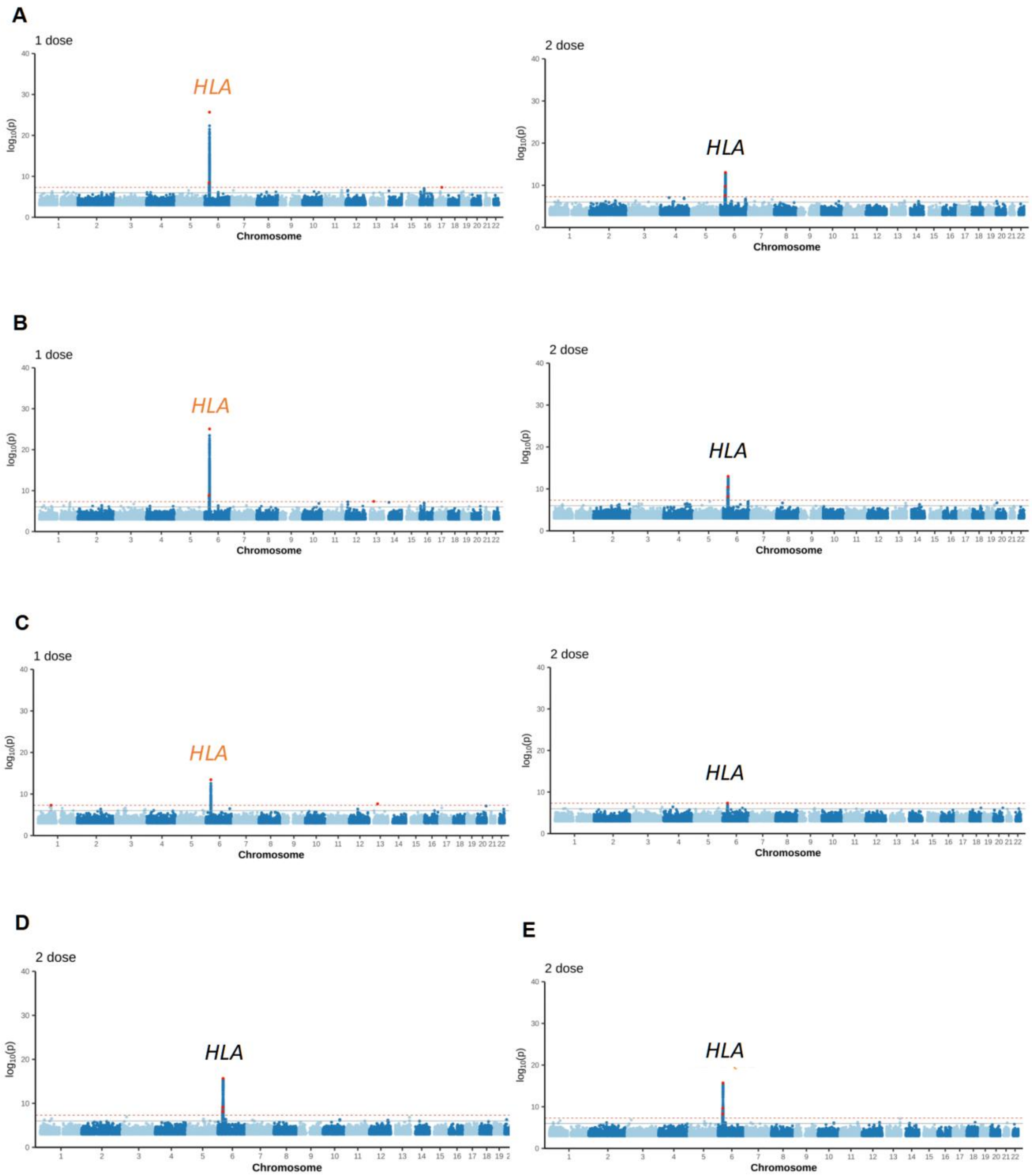

Figure S8-The sensitivity analysis of genome-wide association for host response to COVID-19 vaccine in combined cohorts. (A) The sensitivity analysis that restricted the age to 40-64 years for first-dose and second-dose vaccination cohorts. The red dots represent the lead SNPs with a MAF > 5% within 500kbp blocks. The labeled symbols correspond to the nearest genes or regions of the lead SNPs. If the lead SNP is replicated in the other two cohorts, the font color is red; otherwise, it is black. The red horizontal line corresponds to the genome-wide significance threshold P-value ≤ 5e-8, and the blue horizontal line represents the genome-wide suggestive significance threshold P-value ≤ 1e-6 **(B)** The sensitivity analysis with the time between vaccination and antibody test as covariate for first-dose and second-dose vaccination cohorts. **(C)** The sensitivity analysis with the vaccine type as covariate for first-dose and second-dose vaccination cohorts. **(D)** The sensitivity analysis that

restricted the time between vaccination and antibody test to 7-210 days and added it as covariate for second-dose vaccination cohorts. **(E)** The sensitivity analysis that restricted the time between vaccination and antibody test to 7-210 days for second-dose vaccination cohorts.

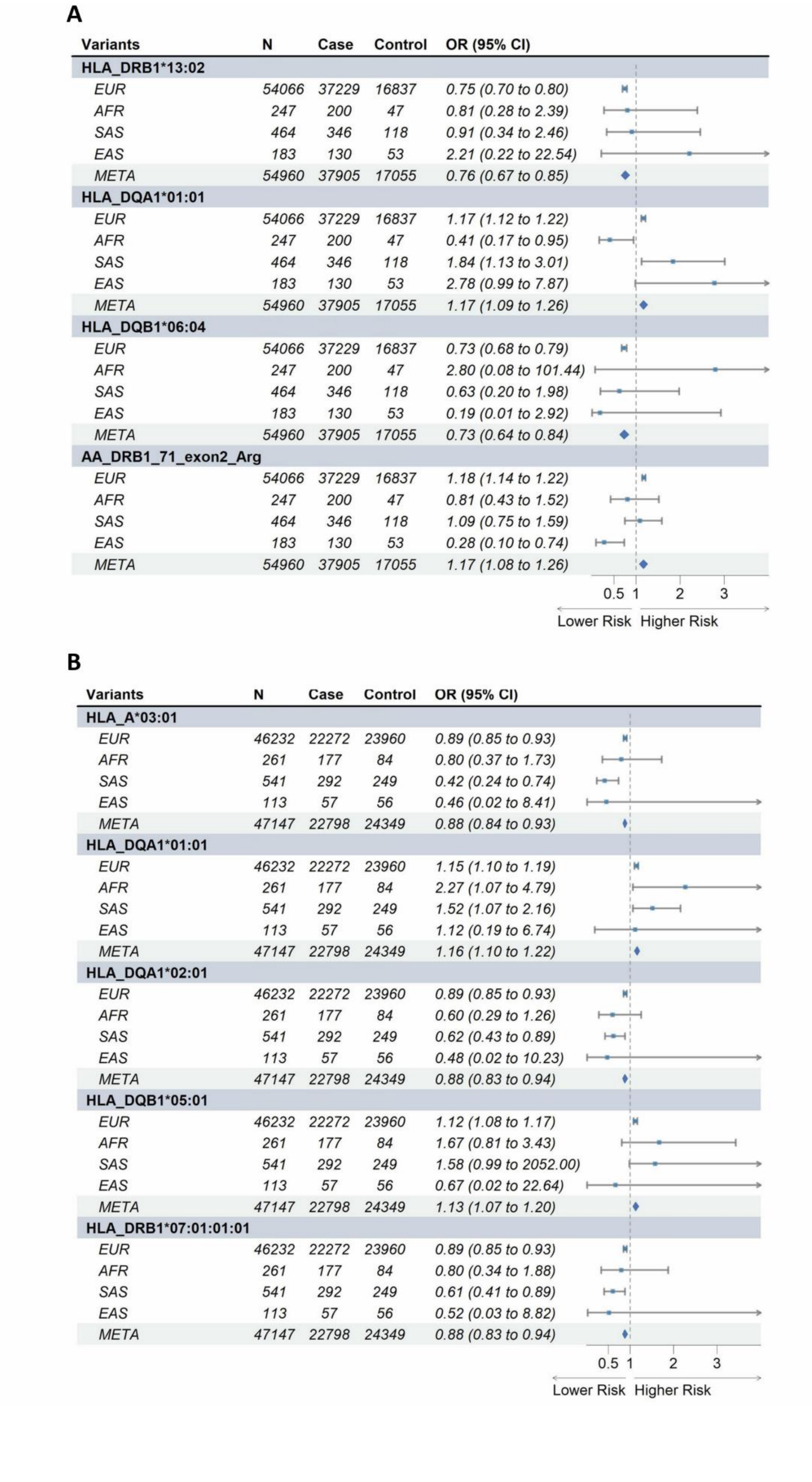

Figure S9-The forest plot of alleles/AAs. (A) The forest plot of three significantly associated alleles and one marginally significantly associated AA with serostatus after first-dose vaccination in cross-ancestry meta analysis. **(B)** The forest plot of five significantly associated alleles with serostatus after second-dose vaccination in cross-ancestry meta analysis.

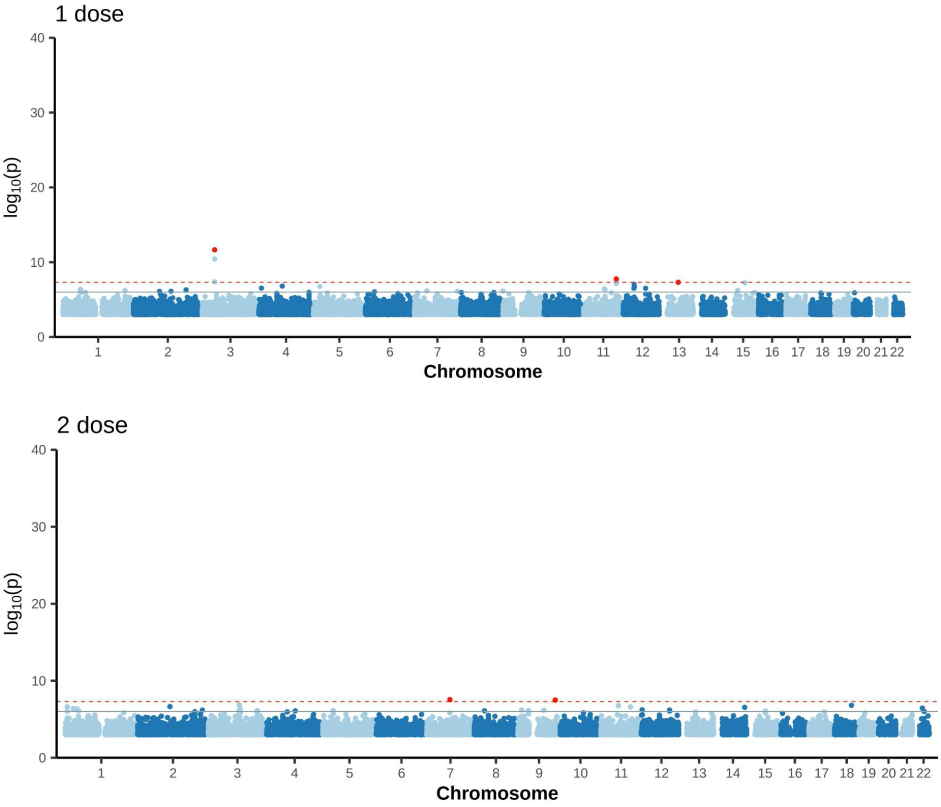

Figure S10-Genome-wide association analysis for the combined cohort comparing the naturally infected population and the uninfected population. The red dots represent the lead SNPs with a MAF > 5% within 500kbp genomic blocks. The red horizontal line corresponds to the genome-wide significance threshold P-value ≤ 5e-8, and the blue horizontal line represents the genome-wide suggestive significance threshold P-value ≤ 1e-6. GWAS were conducted for the first-dose vaccination phenotype antibodies (5,810 naturally infected and 53,889 without natural infection after excluding one, two and third degree genetic relationships) and for the second-dose vaccination phenotype (2,987 naturally infected and 46,164 without natural infection after excluding one, two and third degree genetic relationships), respectively.
